# Platelet-rich therapies in cutaneous and mucocutaneous disorders characterized by inflammatory tissue responses: mechanistic insights and clinical evidence

**DOI:** 10.3389/fmed.2026.1901254

**Published:** 2026-09-18

**Authors:** Eduardo Anitua, Roberto Tierno, Mohammad Hamdan Alkhraisat

**Affiliations:** 1 BTI Biotechnology Institute, Vitoria, Spain; 2 University Institute for Regenerative Medicine and Oral Implantology, UIRMI (UPV/EHU-Fundación Eduardo Anitua), Vitoria, Spain

**Keywords:** epithelial barrier dysfunction, immunomodulation, inflammation, mucocutaneous diseases, platelet-rich derivatives, tissue regeneration

## Abstract

Cutaneous and mucocutaneous dermatoses comprise a heterogeneous group of inflammatory, autoimmune, infectious, and immune-mediated diseases affecting epithelial barrier tissues, including skin and mucosal surfaces. Despite their diverse etiologies these conditions frequently share convergent pathogenic mechanisms characterized by epithelial barrier dysfunction and inflammation, contributing to a substantial global health burden. At the immunopathological level, these disorders frequently involve coordinated activation of innate immune sensing pathways followed by adaptive immune polarization driven by Th1, Th2, Th17 and CD8+ T cell–associated responses. These pathways converge on shared intracellular signaling cascades, resulting in persistent cytokine production, chronic inflammation, and epithelial barrier disruption. Platelet-rich derivatives are autologous biologic therapies enriched in bioactive mediators with pleiotropic regenerative and immunomodulatory properties. Preclinical evidence indicates that these formulations modulate innate immune activation, promote macrophage polarization toward pro-regenerative phenotypes, influence dendritic cell maturation, and support regulatory T cell responses largely via modulation of NF-κB, TLR, PI3K/Akt, MAPK, and TGF-β/SMAD-dependent pathways. In addition to immunomodulation, platelet-rich derivatives enhance tissue repair by promoting cell activation, angiogenesis, and extracellular matrix remodeling. They also contribute to epidermal barrier restoration and normalization of epithelial–immune–microbiome interactions. Despite the heterogeneity of underlying pathogenic molecular pathways, the multi-level effects of platelet-rich formulations provide a rationale for targeting shared downstream processes involved in tissue injury and disease progression. Clinical evidence, although heterogeneous and largely limited to small studies, suggests potential benefit in psoriasis, atopic dermatitis, lichen planus, lichen sclerosus, vitiligo, alopecia areata, and acne, with limited evidence in other mucocutaneous dermatoses associated with substantial primary or secondary inflammation. However, variability in pathophysiological landscapes, platelet-rich formulations and experimental designs limits definitive conclusions. Overall, platelet-derived therapies represent a promising adjunctive approach for restoring immune and epithelial homeostasis in inflammatory mucocutaneous disease, underscoring the need for methodologically standardized clinical and translational research and compliance with minimum reporting standards.

## Introduction

Cutaneous and mucocutaneous dermatoses comprise a heterogeneous group of diseases with variable involvement of skin and mucosal tissues, reflecting different clinicopathological and underlying pathophysiological patterns (1). These conditions may arise from inflammatory, autoimmune, infectious, genetic, or neoplastic mechanisms, and are defined by clinical manifestations affecting the skin and one or more mucosal sites, including oral, genital, ocular, nasal, or gastrointestinal mucosa (2–4), ultimately contributing to a substantial global disease burden driven by chronic morbidity, functional impairment, significant psychosocial impacts, and reduced quality of life (5–9). Among these conditions, clinically relevant inflammation-associated pathogenic processes contributing to tissue injury and clinical phenotype represent a major subgroup that is characterized by convergent inflammatory mechanisms resulting from immune dysregulation or pathogen-induced immune activation (10).

Representative examples include immune-mediated inflammatory cutaneous or mucocutaneous diseases (psoriasis and eczematous dermatitis), interface dermatoses (lichen planus and lichen sclerosus), autoimmune pigmentary and hair follicle–targeted disorders (vitiligo and alopecia areata), inflammatory follicular and adnexal disorders, neurovascular inflammatory dermatoses (rosacea), autoimmune diseases (cutaneous lupus erythematosus, pemphigus vulgaris and bullous pemphigoid), infectious dermatoses (herpes simplex virus infection, candidiasis, impetigo, and secondary syphilis), severe cutaneous adverse reactions (SCARs) (Stevens–Johnson syndrome and toxic epidermal necrolysis) and systemic immune-mediated diseases with cutaneous manifestations (systemic lupus erythematosus, dermatomyositis, and Behçet disease) (4, 11–14). Despite their heterogeneous etiologies, these disorders share convergent pathogenic features characterized by immune dysregulation, epithelial barrier dysfunction, sustained cytokine signaling, and aberrant innate and adaptive immune responses (1, 10, 15–17).

The skin and mucosal surfaces share fundamental structural and immunological features that support the concept of an interconnected epithelial barrier immune network (18–20). Both compartments are composed of specialized epithelial layers populated by resident immune cells, including dendritic cells, macrophages, innate lymphoid cells, and tissue-resident memory T cells, which collectively contribute to immune surveillance, host defense, and maintenance of tissue homeostasis (21–24). Dysregulation of conserved immune pathways, particularly T helper 1, 2, and 17-mediated responses, is a key driver of inflammation across epithelial surfaces, contributing to overlapping cutaneous and mucosal manifestations in immune-mediated inflammatory diseases (25–27). Moreover, beyond their function as structural barrier cells, keratinocytes are increasingly recognized as immunologically active participants in mucocutaneous immunity, contributing to immune surveillance and inflammatory responses through the production of cytokines, chemokines, and antimicrobial peptides, and also via interactions with resident and infiltrating immune cells (28). In fact, epithelial barrier dysfunction, immune dysregulation, and microbiome alterations constitute shared pathogenic mechanisms that link persistent inflammation with impaired tissue repair across epithelial barrier systems (29–32) (Figure 1). Although cutaneous and mucosal compartments retain important anatomical and functional differences, this epithelial barrier framework may provide a mechanistic basis for the multisite involvement observed in mucocutaneous disorders (33).

**Figure 1:**
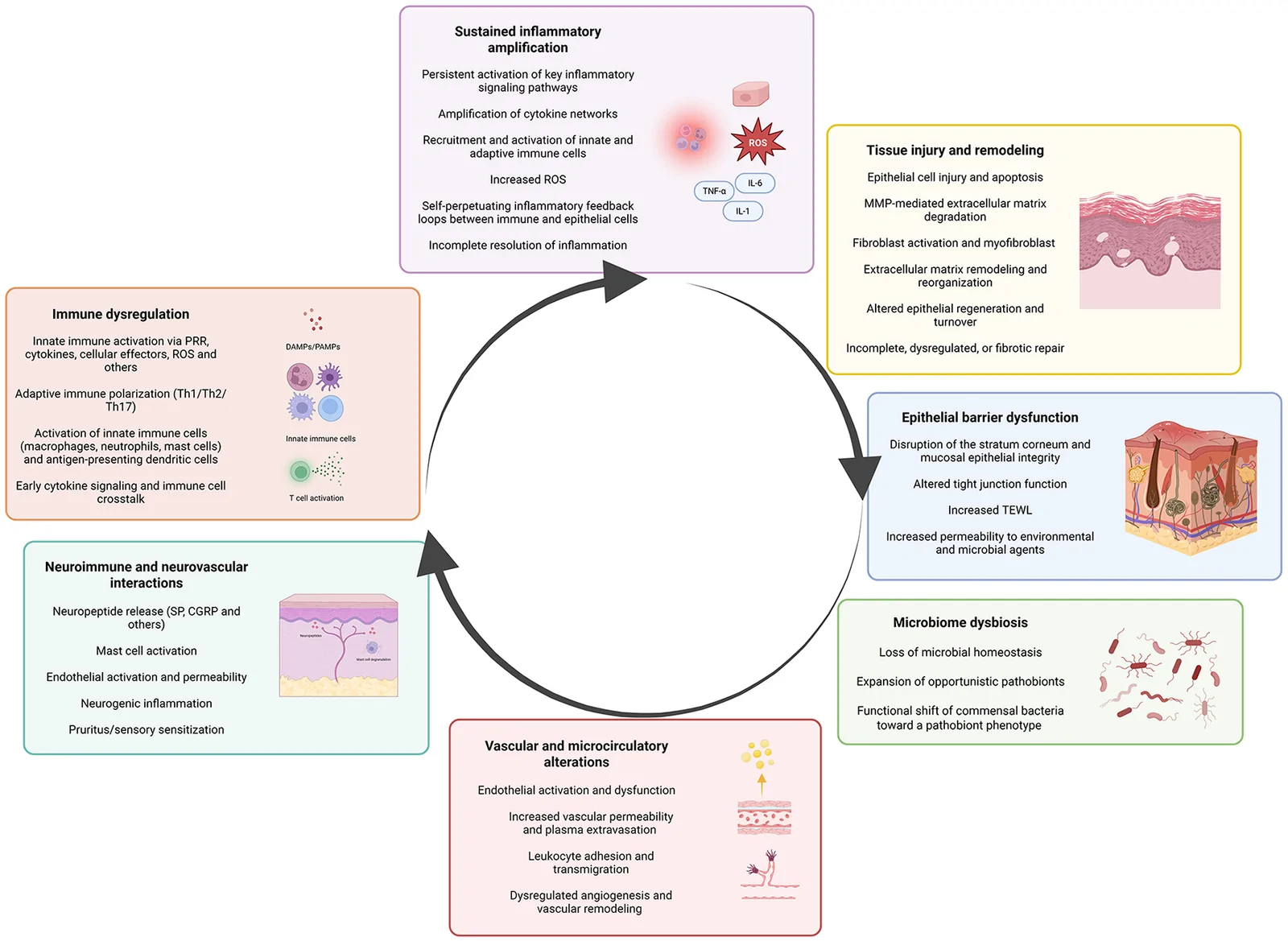
Integrated pathobiological landscape underlying mucocutaneous disorders associated with inflammatory tissue injury and disease progression. PRR, pattern recognition receptors; ROS, reactive oxygen species; Th1, T helper type 1; Th2, T helper type 2; Th17, T helper type 17; SP, substance P; CGRP, calcitonin gene-related peptide; IL-1, interleukin-1; IL-6, interleukin-6; TNF-α, tumor necrosis factor alpha; TEWL, transepidermal water loss. Created with BioRender.com.

Platelet-rich plasma (PRP), including plasma rich in growth factors (PRGF), platelet lysates and releasates have emerged as multifunctional autologous biologics containing a concentrated pool of growth factors and other bioactive molecules (34). Originally developed as regenerative therapies for musculoskeletal and wound-healing applications (35), these preparations are now increasingly explored in dermatology and mucosal immunopathology due to their dual regenerative and immunomodulatory properties (36–39) (Figure 2). From a mechanistic perspective, platelet-rich products exert their biological effects primarily through the release of alpha-granule-associated bioactive mediators such as platelet-derived growth factor (PDGF), transforming growth factor beta (TGF-β), vascular endothelial growth factor (VEGF), epidermal growth factor (EGF), and insulin-like growth factor (IGF-1) (40). Additional effectors include dense granule-derived bioactive components, such as adenosine diphosphate (ADP) and serotonin, antimicrobial peptides, plasmatic proteins, including fibrinogen, fibronectin, vitronectin, complement components, and albumin, platelet-derived extracellular vesicles and platelet-associated calcium ions (41, 42). Collectively, these signaling molecules orchestrate key processes in tissue repair, including cell differentiation, proliferation and migration, angiogenesis, extracellular matrix remodeling and redox modulation (43). However, beyond their classical hemostatic and regenerative roles, platelets are increasingly recognized as key regulators of both innate and adaptive immune responses (44).

**Figure 2:**
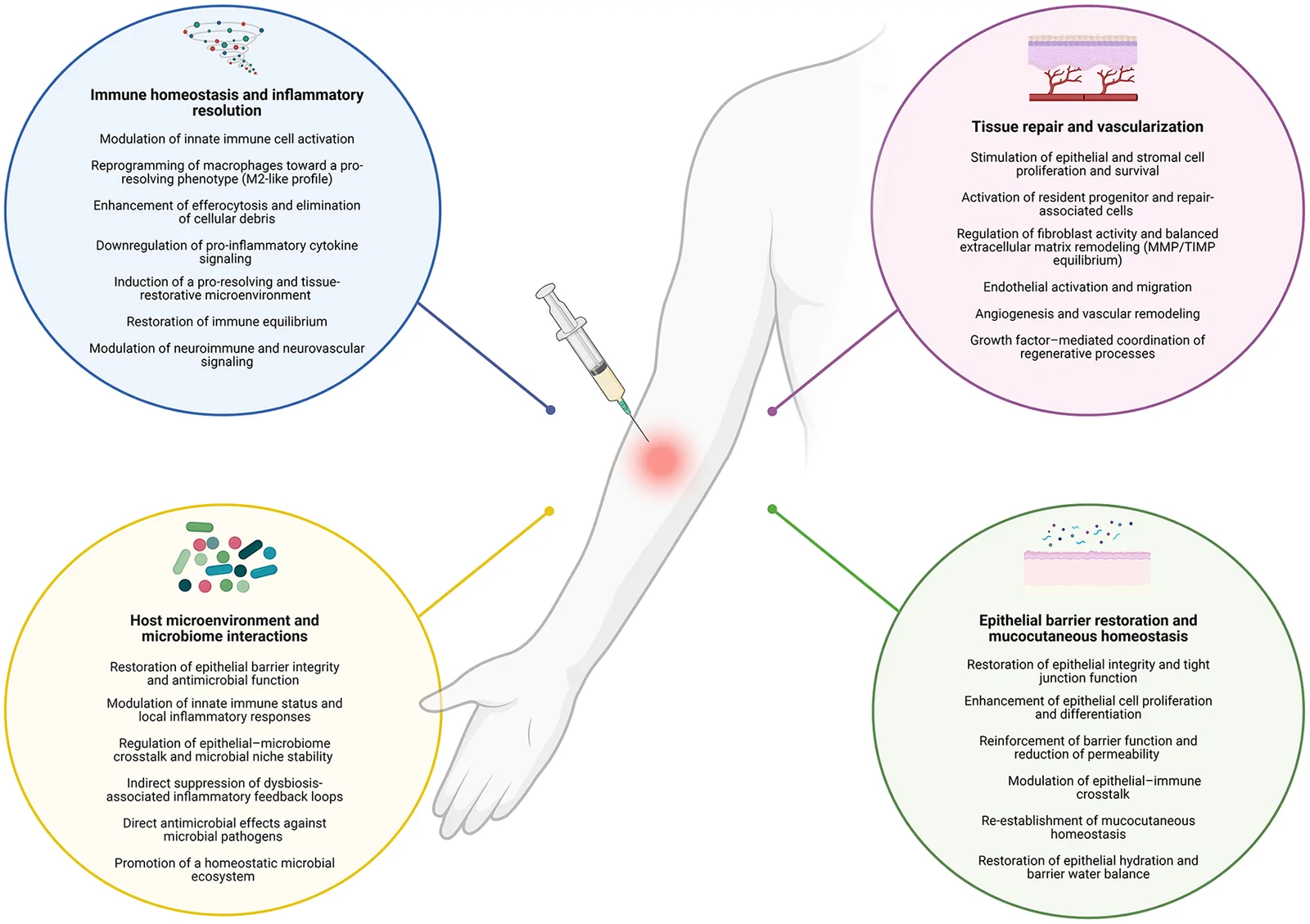
Mechanistic basis of platelet-rich derivative–mediated regenerative and immunomodulatory effects in mucocutaneous conditions. M2, M2 macrophages (alternatively activated macrophages); MMPs, matrix metalloproteinases; TIMPs, tissue inhibitors of metalloproteinases. Created with BioRender.com.

Despite accumulating evidence supporting their regenerative and immunomodulatory properties, the clinical translation of platelet-rich therapies in immune-mediated and/or inflammatory cutaneous and mucocutaneous diseases remains heterogeneous, with variability in preparation methods, cellular composition, and administration protocols contributing to inconsistent outcomes across studies (34, 45–47). This methodological and compositional heterogeneity, together with an incomplete understanding of their immunomodulatory mechanisms in different epithelial microenvironments, underscores the need for a more structured evaluation of their therapeutic potential in immune-mediated and inflammatory epithelial barrier disorders (38, 48). In this context, the present review focuses on cutaneous and mucocutaneous dermatoses in which dysregulated inflammatory or immune-related tissue response contribute to tissue injury and clinical phenotype, and examines the potential role of PRP and other platelet-rich therapies as immunomodulatory effectors. Particular emphasis is placed on disease pathophysiology, key immune and inflammatory pathways, the mechanistic effects of platelet-rich products on tissue repair and immune modulation, and the available clinical evidence. Detailed information on the literature search strategy is provided in Supplementary Note S1.

### Clinical, economic, and psychosocial burden

Cutaneous and mucocutaneous dermatoses in which dysregulated immune-inflammatory programs contribute to tissue injury and shape the pathophenotype represent a substantial and increasing global health burden (8). Despite the fact that most dermatologic conditions are associated with low mortality rates, their high prevalence, chronic or relapsing course, and frequent onset in early life result in a disproportionate impact on long-term morbidity, functional impairment, and quality of life (6). Beyond their clinical burden, chronic dermatoses are increasingly recognized as significant drivers of healthcare expenditure and broader socioeconomic burden. For example, in Europe, moderate-to-severe atopic dermatitis is estimated to generate an annual societal cost of approximately €30 billion with additional out-of-pocket expenditures for patients estimated at €927 annually (49, 50), whereas the mean annual costs of psoriasis and lichen planus was heterogeneous and varied between €1,340 and €8,254, and from €681 to €1,559, respectively (51, 52). This impact arises not only from sustained healthcare utilization and the high costs associated with biologic therapies, but also from substantial indirect costs, including reduced work productivity, psychosocial morbidity, and long-term deterioration in quality of life. Furthermore, the burden of dermatologic disease is not homogeneously distributed across populations, with children and older adults showing greater susceptibility and disease impact, women being disproportionately affected under certain conditions in terms of both prevalence and psychosocial burden, and socioeconomic status as an effect modifier of disease incidence, treatment access, and prognosis (53–61).

Among these conditions, psoriasis and atopic dermatitis are major contributors to the global burden of cutaneous disease. Psoriasis is a chronic immune-mediated inflammatory skin disease characterized by epidermal hyperproliferation, abnormal keratinocyte differentiation, and sustained immune activation (62). This dysregulated cytokine cascade sustains a self-amplifying inflammatory loop that promotes keratinocyte proliferation, impaired differentiation, and further immune cell recruitment, ultimately resulting in the formation of characteristic erythematous scaly plaques (63, 64). Psoriasis constitutes a major global health burden, with an estimated prevalence of approximately 125 million individuals worldwide and affecting around 2–4% of the population in Western countries (65, 66). In addition to its cutaneous manifestations, psoriasis is increasingly recognized as a systemic inflammatory disorder associated with important comorbidities, including psoriatic arthritis, metabolic syndrome, and increased cardiovascular risk (67–69).

Another prominent condition is atopic dermatitis, which represents the most prevalent and clinically significant form of chronic dermatitis worldwide, affecting more than 9% of the global population and accounting for the majority of eczema cases across both pediatric and adult populations (70, 71). Clinically, atopic dermatitis is associated with substantial morbidity, including sleep disturbance, reduced quality of life, and psichological impairment (72, 73). This is a consequence of a distinctive immunological profile that leads to sustained barrier disruption, intense pruritus, and chronic inflammatory amplification, establishing a self-perpetuating disease cycle (74). As reported in patients with psoriasis, this condition has been associated with a range of atopic comorbidities, including asthma, allergic rhinitis, and food allergy, as well as non-atopic comorbidities involving ocular, psychiatric, infectious, endocrine, autoimmune, and cardiovascular systems, in addition to an increased risk of specific malignancies (75). Its epidemiological relevance is supported by its well-characterized pathophysiology, which integrates genetic predisposition affecting epidermal barrier integrity, particularly filaggrin dysfunction, with environmental triggers such as allergens, irritants, microbial dysbiosis, and climatic factors (76). Other relevant clinical subtypes of dermatitis with significant prevalence and impact on health-related quality of life include seborrheic dermatitis (4.4%) (77), nummular dermatitis (0.1–9%) (78) and radiation dermatitis, affecting to some degree up to more than 80% of patients receiving radiotherapy (79–81).

Among dermatoses with lower incidence rates, lichen planus emerges as a chronic inflammatory disorder affecting skin, mucosa, nails, and hair follicles driven predominantly by cytotoxic T-cell–mediated injury to basal keratinocytes, with an estimated prevalence of approximately 0.15–1.5% (82, 83). It is clinically defined by pruritic violaceous papules and plaques and, in mucosal variants, erosive disease associated with significant functional impairment and reduced health-related quality of life (84), and it has been demonstrated to be correlated with a range of systemic pathologies including diabetes mellitus, hypertension, metabolic syndrome, thyroid diseases, psychosomatic disorders, chronic liver disease, gastrointestinal diseases, and genetic susceptibility to cancer (85). Similarly, lichen sclerosus is a chronic inflammatory dermatosis predominantly involving anogenital skin and mucosa, characterized by epithelial atrophy, dermal sclerosis, and lymphocyte-mediated interface inflammation. Despite lower incidence in the general population 0.1–1.7%, it is associated with substantial morbidity due to chronic pruritus, pain, and dyspareunia, with relevant functional and psychosocial impact, particularly in relation to sexuality (86). In addition, long-standing disease is asociated with an increased risk of squamous cell carcinoma, underscoring its clinical relevance and need for long-term monitoring (87).

Infectious dermatoses such as Herpes simplex infection, candidiasis, impetigo, and syphilis constitute a substantial global health burden due to their high prevalence, chronic or recurrent nature, and associated psychosocial and economic consequences (88–91). Beyond direct cutaneous manifestations, these conditions are associated with recurrent healthcare utilization, antimicrobial consumption, stigma, impaired sexual and psychosocial wellbeing, reduced productivity, and increased risk of systemic complications, thereby contributing significantly to both healthcare expenditure and quality-of-life impairment (92). On the other hand, SCARs, including Stevens–Johnson syndrome and toxic epidermal necrolysis, represent the most extreme manifestations of epidermal immune-mediated injury, most commonly resulting from drug-induced hypersensitivity reactions, and are characterized by extensive keratinocyte apoptosis, epidermal detachment, and high acute mortality requiring intensive care management (93). With an estimated global incidence at approximately 0.4–1.2 cases per million individuals per year, reported mortality rates range from 14% to 70%, depending on the clinical phenotype (94).

Within the spectrum of cutaneous and mucocutaneous diseases involving a significant immune-inflammatory component, autoimmune conditions represent a clinically distinct subgroup characterized by low prevalence but high individual disease burden. In this context, autoimmune blistering diseases (AIBDs), including pemphigus vulgaris and pemphigoid diseases, are rare, chronic, and potentially life-threatening immune-mediated disorders driven by autoantibody-mediated loss of epithelial adhesion, resulting in mucocutaneous blistering, pain, secondary infection risk, substantial treatment-related morbidity, psychosocial impairment and relativelly frequent neurological comorbidities (95–97). Bullous pemphigoid represents the most common AIBD in older adults and is associated with increased mortality, largely driven by age-related comorbidities and complications of immunosuppressive therapy (98). These conditions have estimated incidences ranging from approximately 0.4–30 cases per million persons per year for pemphigus vulgaris and 1–48 cases per million persons per year for bullous pemphigoid, with a marked age-dependent increase in incidence in older populations (99). Regarding cutaneous lupus erythematosus, this chronic autoimmune dermatosis is associated with photosensitive lesions, frequent scarring, and a significant long-term burden derived from chronicity, relapse, coexisting autoimmune comorbidities and reduced health-related quality of life (100, 101), with an estimated incidence rate of 3.9 cases per 100,000 individuals (102).

In addition, a major group of cutaneous and mucocutaneous diseases with a significant immune-inflammatory component are characterized by low mortality but a disproportionate burden on health-related quality of life and healthcare utilization, largely driven by chronicity, visibility, pain, and substantial psychosocial impact (103). This impact is commonly expressed through impaired self-esteem, anxiety, depression, social isolation, and disruption of interpersonal, occupational, and daily functioning, reflecting the central role of skin appearance and symptom visibility in patient well-being (104–106). The group includes highly prevalent conditions such as acne vulgaris (85% of young adults aged 12–25 years) (107), and rosacea (5% in the general population), in which facial involvement and chronic relapsing symptoms are key determinants of psychosocial burden (108, 109). Lower-prevalence high-impact disorders such as hidradenitis suppurativa (0.7–1.5%) (110), characterized by recurrent painful nodules, abscesses, sinus tract formation, and scarring in intertriginous areas, are associated with severe pain, functional limitation, and reduced productivity, with significant impacts on mental health and social functioning (111). Similarly, vitiligo (0.4% in the general population) (112) and alopecia areata (2% in the general population) (113) are associated with a substantial psychosocial morbidity resulting from visible phenotypic changes, stigma, and reduced quality of life, an also with the incidence of a range of autoimmune and psychiatric comorbidities (114, 115). Infectious dermatoses further contribute to healthcare utilization and psychosocial burden, largely due to recurrence, visibility, and cosmetic impact rather than systemic disease severity.

### Immunopathogenic mechanisms

From a pathophysiological perspective, cutaneous and mucocutaneous dermatoses in which inflammatory or immune-related tissue responses contribute to tissue injury and clinical phenotype do not arise from a single etiological origin (16). However, they frequently exhibit substantial convergence in downstream immunopathological pathways characterized by dysregulated innate and adaptive immune responses (16), sustained cytokine signaling, and activation of common downstream inflammatory mediators, including tumor necrosis factor alpha (TNF-α), interleukin 1 (IL-1) family cytokines, and intracellular signaling cascades such as nuclear factor-κB (NF-κB) and Janus kinase/signal transducer and activator of transcription (JAK/STAT) (15, 116, 117), although the relative contribution of each axis varies according to the specific disease context (65, 76, 118–123). At the tissue level, such mechanisms underlie distinct clinicopathological phenotypes, in which the predominant immune effector mechanisms and target tissue compartment determine the clinical presentation, including erythema, scaling, pruritus, erosions, or sclerosis, depending on whether inflammation primarily affects the epidermis, dermoepidermal junction, or dermal stroma (16, 124). Within this broader inflammatory spectrum, cutaneous immune-mediated diseases represent distinct immunological endotypes characterized by divergent upstream immune polarization, but partially converging downstream inflammatory signaling pathways (125). Nevertheless, considering the substantial heterogeneity in the pathophysiological mechanisms underlying cutaneous and mucocutaneous disorders, these conditions are better understood as mechanistically convergent endotypes rather than etiologically distinct entities, reflecting shared downstream inflammatory architectures across clinically heterogeneous diseases.

### Pattern recognition and innate immune activation

Innate immune sensing represents the primary initiating component of cutaneous and mucosal inflammation, connecting environmental triggers and epithelial stress to activation of conserved inflammatory programs (126). These responses are triggered by tissue damage and barrier disruption and mediated through pattern recognition receptor (PRR) sensors and other complementary innate sensing pathways distributed across cutaneous cell populations, including keratinocytes, Langerhans cells, stromal and endothelial cells, macrophages, and other tissue-resident immune cells (127, 128). This class of germ line-encoded receptors, including Toll-like receptors (TLR), nucleotide-binding oligomerization domain (NOD)-like receptors, and retinoic acid–inducible gene I (RIG-I)–like receptors exerts a key sensing function for pathogen-associated (PAMPs) and endogenous damage-associated molecular patterns (DAMPs) (129). Following PRR activation, epithelial barrier dysfunction and persistent tissue stress amplify innate immune responses via inflammasome-driven cytokine maturation and complement-mediated inflammatory amplification, thus propagating and sustaining early inflammatory responses (126, 130, 131).

This immune axis is particularly relevant in dermatoses in which microbial exposure or dysbiosis constitutes the dominant initiating factor (17). In infectious dermatoses characterized by prominent host inflammatory responses, including herpes simplex virus infection, candidiasis, impetigo, and secondary syphilis, PRR engagement triggers pathogen-specific but mechanistically convergent inflammatory programs (10). These responses are characterized by type I interferon–driven antiviral immunity in viral infections, Th17-driven neutrophil-associated responses in fungal disease, and mixed innate–adaptive immune activation in bacterial and spirochetal infections, ultimately resulting in epidermal and dermal inflammation of variable intensity and pattern (26, 127, 132, 133). A similar principle applies to non-infectious conditions in which innate immune activation occurs in a context of sterile or microbiome-associated inflammation. Across these conditions, innate immune activation acts as an upstream driver of inflammatory intensity, shaping both the magnitude and qualitative nature of downstream adaptive immune responses (134).

Inflammatory acne vulgaris exemplifies this paradigm, where *Cutibacterium acnes*–associated dysbiosis, follicular hyperkeratinization, and an altered sebaceous microenvironment contribute to Toll-like receptor 2 (TLR2) activation and inflammasome-mediated interleukin 1β (IL-1β) production (135, 136). These pathways promote a predominantly neutrophilic inflammatory response within the pilosebaceous unit, which may progress to follicular rupture and extension of inflammation into the adjacent dermis (137). In addition, these epithelial innate sensing pathways are also involved in sterile inflammatory dermatoses, including psoriasis, atopic dermatitis, and interface disorders (65, 138–141). In these diseases, endogenous DAMPs, epithelial stress, and barrier dysfunction activate convergent PRR, inflammasome-, and cytokine-driven pathways, resulting in sustained inflammatory amplification in the absence infection (128).

### Adaptive immune effector pathways

Immune-mediated inflammatory dermatoses are governed by highly integrated immune effector circuits that couple innate immune sensing to adaptive immune polarization and cytokine-driven tissue remodeling (126). Rather than representing isolated inflammatory pathways, these networks include dynamic interactions between tissue-resident cells, antigen-presenting cells, innate lymphoid populations, and polarized T cell subsets that collectively establish disease-specific inflammatory endotypes (142–144). Chronic inflammatory dermatoses are broadly characterized by dominant Th1-, Th2-, Th17-, and CD8-positive T lymphocyte (CD8+ T cell)–associated immune axes that modulate disease pathogenesis, with substantial convergence at common transcriptional and signaling hubs including NF-κB, JAK/STAT, activator protein 1 (AP-1) and signal transducer and activator of transcription 3 (STAT3) pathways (145–148). These integrated circuits ultimately determine keratinocyte behavior, barrier integrity, leukocyte recruitment, neuroimmune activation, and chronic tissue remodeling (149–151).

Adaptive immune responses sustain chronicity and define tissue-specific inflammatory phenotypes across multiple dermatoses (152). At the immunopathological level, epidermal-dominant inflammatory dermatoses can be broadly categorized into specific immune endotypes, with psoriasis and eczematous dermatoses representing prototypical interleukin 23 (IL-23)/Th17- and type 2–skewed inflammatory spectra, respectively (125). In particular, psoriasis is sustained by a dysregulated crosstalk between innate and adaptive immune responses, with activation of dendritic cells and T lymphocytes leading to overproduction of pro-inflammatory cytokines, including TNF-α, interleukins IL-1, 17 (IL-17), 19 (IL-19), 22 (IL-22), 23 (IL-23) and 36 (IL-36) sub-family cytokines, largely sustained by NF-κB and STAT3-dependent signaling pathways (153, 154), together with dysregulated keratinocyte growth factor signaling contributing to epidermal hyperproliferation and impaired differentiation (155). Conversely, eczematous dermatoses comprise a heterogeneous group of inflammatory conditions characterized by different upstream pathogenic mechanisms, including immune dysregulation, epidermal barrier dysfunction, microbial interactions, and environmental triggers, converging on shared downstream programs of epidermal inflammation and barrier impairment (156).

Within this spectrum, atopic dermatitis represents the most prevalent chronic inflammatory dermatosis worldwide, particularly in childhood, and constitutes the best-characterized model of type 2–skewed cutaneous inflammation (157). It is predominantly driven by T helper 2 (Th2)-polarized immune responses and overexpression of cytokines including interleukins 4 (IL-4), 13 (IL-13), and 31 (IL-31), alongside contributions from Th22 and, in chronic stages, Th1-associated pathways (75–78). In this context, IL-4 and IL-13–driven type 2 signaling is associated with epidermal barrier dysfunction, including aberrant keratinocyte differentiation, altered lipid metabolism, impaired tight junction integrity, and also with downregulated filaggrin expression in some patients (158–160). This is also accompanied by IL-31–mediated pruritus, which sustains chronic itch and reinforces itch–scratch cycles that perpetuate inflammation (161, 162). Moreover, interleukin 5 (IL-5) contributes primarily via amplification of eosinophilic inflammation, supporting systemic and tissue eosinophilia in specific disease endotypes (163). In the same line, scleroderma pathogenesis involves a Th2-skewed immune response characterized by IL-4 and IL-13–driven activation of fibroblasts, macrophage and fibroblast production of transforming growth factor beta (TGF-β), enhanced collagen synthesis, and recruitment of additional immune cells, with contributory roles from B cells, mast cells, and potentially Th17 cells in sustaining inflammation and fibrosis (124).

Cytotoxic adaptive immunity constitutes a central effector module in interface and pigmentary dermatoses, where tissue-specific immune injury derives from integrated CD8+ T cell–driven inflammatory circuits embedded within Th1-skewed and interferon gamma (IFN-γ)–dominant immune microenvironments sustained by reciprocal interactions between cytotoxic lymphocytes, antigen-presenting cells, and tissue-resident epithelial or stromal cells (164–166). In lichen planus, CD8+ T cell–mediated cytotoxic immune responses promote basal keratinocyte apoptosis through canonical effector pathways, including perforin/granzyme release and Fas (CD95)/Fas ligand (FasL) signaling pathway (Fas–FasL), within a chemokine-rich inflammatory niche characterized by IFN-γ-induced C-X-C motif chemokine receptor 3 (CXCR3) ligand expression and sustained antigen presentation by local tissue and antigen-presenting cells (167–169). A particular case of this CD8+ T cell–driven immunopathogenic paradigm is observed in lichen planopilaris, which exhibits IFN-γ-JAK/STAT signaling activation and C-X-C motif chemokine ligand 9/10 (CXCL9/CXCL10)-mediated recruitment of CXCR3+ Th1/type 1 cytotoxic T cells (Tc1) following collapse of follicular immune privilege. This situation leads to irreversible destruction of the follicular stem cell niche and progressive cicatricial alopecia driven by chronic lichenoid inflammation and subsequent fibrosis (170, 171).

In vitiligo, melanocyte loss is mediated by autoreactive CD8+ T cells, including tissue-resident memory and recirculating effector subsets, whose persistence is sustained by an IFN-γ-dependent chemokine amplification loop. This loop is characterized by induction of CXCL9 and CXCL10 in keratinocytes and myeloid cells, promoting CXCR3-mediated recruitment and retention of effector T cells within lesional skin (172–174). Collectively, these interactions result in a self-sustaining interferon–JAK/STAT signaling circuit that integrates adaptive immune activation with epithelial and stromal reprogramming, thereby reinforcing local immune memory and melanocyte immune susceptibility (175). Conversely, lichen sclerosus represents a chronic fibroinflammatory dermatosis with predominant dermal involvement, characterized by progressive sclerosis, hyalinisation, and architectural remodeling of the extracellular matrix (176). Although its immunopathogenesis is not fully elucidated, it is considered a chronic immune-mediated dermatosis characterized by Th1-polarized inflammation, aberrant extracellular matrix remodeling, and progressive dermal fibrosis (86). To complete the overview of lichenoid dermatoses, cutaneous lupus erythematosus exemplifies an interferon-driven interface and dermal immune complex–mediated disease, with prominent type I interferon signaling and complement activation contributing to tissue injury and scarring phenotypes (177).

On the other hand, alopecia areata exhibits key immunopathological parallels with vitiligo, reflecting a shared cytotoxic CD8+ T cell–driven inflammatory response (178). It results from collapse of hair follicle immune privilege, characterized by IFN-γ-driven upregulation of major histocompatibility complex (MHC) class I expression, dysregulated antigen presentation, and local immune activation within the hair follicle (179). This is accompanied by infiltration of cytotoxic CD8+ T cell subsets, including natural killer group 2, member D (NKG2D)-expressing effector memory populations, which mediate targeted damage to follicular architecture (180). Disease persistence is sustained by a cytokine-rich microenvironment dominated by IFN-γ and interleukin 15 (IL-15) signaling, which activates downstream JAK/STAT pathways, reinforcing antigen presentation, immune cell recruitment, and chronic cytotoxic effector activity (181–183).

### Humoral autoimmune effector mechanisms

Autoimmune conditions affecting the skin and mucous membranes represent a highly diverse group of disorders involving specific structural, vascular, and immunologically active compartments of epithelial tissues (184). Within this continuum, autoimmune blistering diseases represent a prototypical antibody-mediated epithelial adhesion pathology (185). AIBDs, including pemphigus and pemphigoid disorders, are mediated by pathogenic autoantibodies directed against distinct components of epidermal and dermal–epidermal adhesion complexes (186). Pemphigus vulgaris and related pemphigus variants are characterized by immunoglobulin G (15) autoantibodies targeting desmoglein 3 and desmoglein 1 within desmosomes, resulting in proggressive acantholysis and intraepidermal blister formation (187). In contrast, pemphigoid diseases, including bullous pemphigoid, are driven by autoantibodies against hemidesmosomal proteins such as bullous pemphigoid antigens 180 (BP180) and 230 (BP230), leading to complement activation and fragment cristalizable (Fc) receptor–mediated recruitment of inflammatory cells, particularly eosinophils and neutrophils, culminating in subepidermal blister formation (188). These pathogenic processes are further amplified by complement cascade activation and protease release, which contribute to dermal–epidermal junction disruption and tissue injury (95).

On the other hand, cutaneous lupus erythematosus exemplifies immune complex–driven interface dermatitis, characterized by deposition of immune complexes at the dermoepidermal junction, complement activation, and a prominent type I interferon–skewed inflammatory signature (189, 190). Clinically, this results in photosensitive inflammatory lesions with basal keratinocyte damage, vacuolar interface changes, and variable progression toward dermal inflammation and scarring depending on subtype and chronicity (191). Beyond these prototypical conditions, other clinically relevant autoimmune and immune-mediated dermatoses include dermatomyositis, defined by complement-mediated microvascular injury and type I interferon activation, localized scleroderma, associated with immune-driven fibroblast activation and dermal fibrosis, and vasculitic syndromes, in which immune complex deposition and neutrophil-mediated vascular injury lead to purpura and ulceration (192–194).

### Barrier dysfunction and epithelial stress responses

Sustained inflammation across cutaneous and mucosal tissues frequently converges on epithelial barrier dysfunction and stress-associated remodeling responses that perpetuate chronic inflammation and defective tissue repair (195). In most eczematous dermatoses, alterations in keratinocyte differentiation, lipid composition, and tight junction compromise barrier function integrity, thus facilitating chronic immune activation (196, 197). In other dermatoses, including psoriasis, pemphigus and pemphigoid diseases, infectious dermatoses, lichen planus and other lichenoid disorders, epithelial barrier dysfunction is predominantly a consequence of persistent inflammation, immune-mediated tissue injury or pathogen-driven ephitelial damage, reflecting a secondary self-perpetuating amplification loop that further sustains disease chronicity (29, 185, 198–200). In follicular disorders such as acne and hidradenitis suppurativa, structural disruption of the pilosebaceous unit leads to exposure of the surrounding dermal milieu to microbial products and inflammatory mediators, thus amplifying tissue injury and chronicity (137, 201).

### Microbiome interactions

Microbiome–host interactions constitute a dynamic modulatory layer of cutaneous and mucosal immunity (202). In atopic dermatitis, immune–epithelial interactions are further shaped by cutaneous microbiome dysbiosis (203). In particular, *Staphylococcus aureus* colonization is associated with epidermal barrier disruption and contributes to amplification of inflammatory responses and further barrier impairment (204, 205). In psoriasis, increased bacterial burden, reduced microbial diversity, and a particular compositional shift characterized by enrichment of *Staphylococcus* and *Corynebacterium*, together with depletion of commensal taxa such as *Cutibacterium* have been reported in psoriatic lesions (206). In the same line, specific dysbiotic patterns have been identified across several dermatoses, including lichenoid disorders (207–210), acne vulgaris (211), phemfigous and phemfigoid diseases (212), alopecia areata (213, 214) or hidradenitis suppurativa (215, 216), underscoring the role of the microbiome in disease pathophysiology. In follicular disorders such as inflammatory acne, *Cutibacterium acnes*–associated dysbiosis triggers innate immune activation within the pilosebaceous unit (217). At mucosal surfaces, *Candida* species and other commensals may transition toward pathogenic behavior under conditions of immune dysregulation, reinforcing IL-17–mediated inflammatory pathways and sustaining tissue inflammation (218). Furthermore, mucosal bacteria community structure and diversity can also modulate *Candida albicans* virulence, with changes in *Enterococcus* and *Lactobacillus* abundance contributing to alterations in fungal pathogenic potential (219).

### Neuroimmune and neurovascular crosstalk

Neuroimmune and neurovascular interactions represent an additional axis shaping cutaneous inflammation, particularly in conditions characterized by dysregulated sensory and vascular responses (220). Bidirectional interactions between sensory neurons, keratinocytes, endothelial cells, mast cells, and resident immune populations contribute to inflammatory amplification via neuropeptides, cytokines, and vasoactive mediators (221). In particular, substance P, calcitonin gene-related peptide (CGRP), and transient receptor potential vanilloid 1 (TRPV1)-dependent signaling promote vasodilation, leukocyte recruitment, pruritus, and neurogenic inflammation, thus contributing to chronic inflammatory responses and epithelial barrier dysfunction in inflammatory mucocutaneous disorders (222). Rosacea exemplifies this mechanism, with TLR2 overactivation, increased cathelicidin (LL-37) processing, and kallikrein-mediated inflammatory amplification contributing to persistent erythema, flushing, and papulopustular inflammation (122, 223). In atopic dermatitis, neuroimmune signaling is further amplified through IL-31–mediated pathways, which directly activate sensory neurons and reinforce itch–scratch cycles, thereby perpetuating epithelial damage and chronic inflammation (161). The role of TRPV1 expression in cutaneous homeostasis was further confirmed by Ehnis-Pérez et al. (224), with evidence of its upregulation in individuals with sensitive skin.

## SCARs

In parallel, SCARs such as Stevens–Johnson syndrome and toxic epidermal necrolysis represent life-threatening, drug- or infection-associated hypersensitivity reactions characterized by massive keratinocyte apoptosis driven predominantly by cytotoxic CD8+ T cells and natural killer (NK) cells (225, 226). Rather than a single cytotoxic pathway, epithelial injury is mediated by a coordinated network of effector molecules, including perforin/granzyme B, Fas–FasL interactions, and TNF-family death ligands; however, accumulating evidence identifies secretory granulysin as the dominant cytotoxic mediator in lesion fluids, strongly correlating with disease severity and sufficient to reproduce Stevens–Johnson syndrome/toxic epidermal necrolysis -like epidermal necrosis in experimental models. These processes culminate in full-thickness epidermal necrosis, dermoepidermal separation, and extensive mucocutaneous detachment (227).

### Preclinical evidence

#### Immune homeostasis and inflammatory resolution

Upon activation, platelets release a broad repertoire of bioactive molecules, including growth factors, cytokines, chemokines, antimicrobial peptides, and extracellular vesicles, which collectively modulate inflammation, tissue repair, angiogenesis, and immune responses (228). At a first level of regulation, actions exerted by platelet derivatives are mediated by chemokines such as platelet factor 4 (PF4/CXCL4), C-C motif chemokine ligand 5 (CCL5/RANTES), and C-X-C motif chemokine ligand 7 (CXCL7), which rapidly modulate leukocyte recruitment dynamics following local exposure (229, 230). These mediators interact with endothelial cells and circulating leukocytes, modulating adhesion, chemotaxis, and tissue infiltration patterns (231). C-X-C motif chemokine ligand 12 (CXCL12) further contributes by promoting progenitor cell recruitment and tissue-resident repair programs (232, 233). These early events develop within a context of sterile tissue injury predominantly driven by DAMP, whereas under certain circumstances PAMPs may also contribute to pattern recognition receptor activation, including TLR-dependent signaling pathways (234). Once immune cells are recruited into an environment enriched in platelet-rich effectors, their functional phenotype is shaped by the combined influence of bioactive molecules and the altered cytokine milieu. Rather than directly enforcing macrophage polarization or dendritic cell maturation, platelet derivatives modulate the local signaling context, including growth factor receptor activation, altered costimulatory balance, and changes in local cellular stress signals (235).

In this context, platelet derivatives modulate innate immune responsiveness to DAMP-driven signals and downstream pattern recognition receptor–associated pathways by integrating platelet-derived chemokines, growth factors, and vesicle-associated mediators that regulate signaling thresholds in pattern recognition receptor–expressing cells (236, 237). This involves context-dependent adjustments in signal transduction strength and duration in terms of TLR-dependent priming, NOD-like receptor family pyrin domain containing 3 (NLRP3) inflammasome activation, and downstream NF-κB and mitogen-activated protein kinase (MAPK) pathways, leading to a modulation of the amplitude and kinetics of inflammatory amplification cascades (238–240). Preclinical *in vitro* studies involving macrophages, and dendritic cells suggest that platelet-rich formulations promote a shift toward a pro-regenerative immune profile rather than simple immunosuppression by inducing M1/M2 macrophage polarization (241–245) and modulating dendritic cell maturation (246–248). These results have been further confirmed using *in vivo* models (249–251).

Platelet-rich formulations may also indirectly support regulatory T-cell responses (252–254). In particular, an increase in regulatory T cells (Treg) populations has been observed in patients with Behçet's disease treated with PRP (255). In this sense, TGF-β1 is considered a central mediator of this immunomodulatory effect, promoting regulatory Treg differentiation and suppressing effector T-cell activation, thus limiting excessive adaptive immune responses (256, 257). Effects of platelet-derived formulations on adaptive immunity are largely mediated through modulation of antigen-presenting cell (APC) function, particularly dendritic cell activation state, costimulatory molecule expression, and cytokine secretion profiles (258). These changes affect the priming conditions of naïve T cells, thus influencing lineage commitment toward Th1-, Th17-, or regulatory phenotypes (235, 259). Experimental studies have further explored the relationship between the exposure to platelet-rich derivatives with altered antigen-presenting cell signaling profiles, including changes in dendritic cell activation state and cytokine production under inflammatory conditions (260). According to different experimental models of mucocutaneous disorders, a relative shift in the inflammatory milieu, characterized by reduced Th1- and Th17-associated signals and an increased expression of regulatory and tissue-reparative cytokines compatible with a type 2–skewed immune profile was observed in response to PRP (254, 261–263). *In vitro* models of dermal fibroblasts and keratynocites exposed to a pro-inflammatory environment have also demonstrated that PRGF mitigates oxidative stress and stimulates cell viability, proliferation and migration rates (264), whereas El Backly et al. observed enhanced wound healing in inflammed keratinocytes in response to platelet lysate (265).

These processes are commonly associated with downregulation of NF-κB–dependent signaling pathways and reduced production of key pro-inflammatory mediators (236, 265–269), potentially mediated via coordinated effects on upstream TLR, interleukin-1 receptor (IL-1R) and tumor necrosis factor receptor (TNFR)-dependent pathways (270, 271), intracellular cross-talk involving Phosphoinositide 3-kinase/ protein kinase B (PI3K/Akt) (272, 273), MAPK (274), and TGF-β/SMAD signaling (275), miRNA-mediated regulation of adaptor proteins or the metastasis-associated lung adenocarcinoma transcript 1/microRNA-217/sirtuin 1 (MALAT1/miR-217/SIRT1) regulatory axis (268, 276), and broader microenvironmental changes, including reduced oxidative stress and macrophage reprogramming toward pro-resolving phenotypes (277–279). Similar trends have been observed in different *in vivo* experimental models (249, 280, 281). Downstream effects often include reduced expression of TNF-α, IL-1β, and interleukin 6 (IL-6) (243, 282–285), and relative upregulation of anti-inflammatory mediators such as interleukin 10 (IL-10) in several models (246, 249, 286–290). These effects appear context-dependent and vary across formulations, experimental conditions and time (291). Particularly, leukocyte content is considered a key determinant of the inflammatory profile of platelet-rich formulations, influencing both the magnitude and the direction of the observed immunomodulatory effects (292, 293).

#### Tissue repair and vascularization

Mucocutaneous diseases are commonly characterized by varying degrees of tissue dysfunction and structural impairment, often driven by dysregulated immune responses and sustained inflammatory activity. In a broad spectrum of inflammatory, immune-mediated, and infectious skin diseases, persistent immune activation contributes to tissue injury, altered cellular and extracellular matrix homeostasis, and disruption of the cutaneous microenvironment (16). *In vitro*, e*x vivo* and *in vivo* wound-healing systems demonstrate that platelet-rich formulations enhance coordinated tissue repair programs (46, 269, 294, 295). These include fibroblast activation and proliferation, keratinocyte migration, and accelerated re-epithelialization, mediated through growth factor–dependent receptor signaling involving PDGF, EGF, TGF-β, and IGF-1 (296). Importantly, this regenerative response is associated to extracellular matrix remodeling, with modulation of extracellular matrix synthesis and degradation (269, 297). Controlled extracellular matrix deposition during cutaneous wound healing is a tightly regulated reparative process that provides provisional structural support and tissue stabilization. However, sustained activation due to persistent inflammation or dysregulated mechanotransduction leads to fibrosis, characterized by progressive matrix accumulation and disruption of tissue architecture and function (298, 299).

Platelet-rich derivatives may differentially modulate Smad2/3 signaling depending on their composition and protocol, which determine the relative bioavailability of profibrotic and antifibrotic mediators (300–302). Central to this process is TGF-β1, which interacts with the TGF-β receptor I/II complex to activate SMAD2/3-dependent transcriptional programs that, in the presence of interleukin-2 (IL-2)/STAT5 signaling, promote forkhead box P3 (FOXP3) expression and epigenetic stabilization of the regulatory T-cell (Treg) lineage (303, 304). Although the TGF-β1/SMAD2/3 pathway represents a central profibrotic signaling axis involved in tissue remodeling and fibrosis, platelet-rich derivatives may also modulate downstream fibrotic responses through growth factor–mediated activation of parallel signaling pathways, including PI3K/Akt and mitogen-activated protein kinase/extracellular signal-regulated kinase (MAPK/ERK) downstream of PDGF receptor, epidermal growth factor receptor (EGFR) and IGF-1 receptor, thus influencing fibroblast activation states and extracellular matrix remodeling dynamics (273, 305–308). Recent literature hypothesizes that this regulation might extend to mechanotransduction pathways, suggesting that PRP could bidirectionally modulate scar formation via the Piezo1-YAP/TAZ axis depending on the mechanical microenvironment (309). In addition, PRP may indirectly affect extracellular matrix composition, including proteoglycans such as decorin, which can modulate TGF-β signaling bioavailability and activity, contributing to a context-dependent regulation of fibrotic vs. regenerative outcomes (273).

Beyond these canonical pathways, platelet derivatives may exert antifibrotic effects through additional complementary mechanisms, including hepatocyte growth factor/ mesenchymal-epithelial transition factor (HGF–c-MET) signaling, which antagonizes TGF-β-driven myofibroblast activation, EGFR-mediated acceleration of re-epithelialization, which may indirectly limit the persistence of pro-fibrotic dermal signaling by reducing the duration of pro-fibrotic signaling activity within the dermal repair microenvironment, or via miRNA-mediated post-transcriptional regulation of collagen synthesis and matrix remodeling pathways (310–313). In parallel, PRP can attenuate oxidative stress–driven amplification of TGF-β signaling, limiting reactive oxygen species (ROS)-mediated fibroblast activation (314, 315), while also regulating matrix metalloproteinases/tissue inhibitors of metalloproteinases (MMP/TIMP) balance to promote controlled extracellular matrix turnover and resolution of fibrotic tissue (316). In addition, platelet derivatives also contribute to tissue repair through coordinated activation of angiogenic and stromal remodeling pathways. Growth factors present in platelet-rich preparations, including VEGF, PDGF, fibroblast growth factor 2 (FGF-2), TGF-β1, hepatocyte growth factor (HGF) and IGF-1, together with pro-angiogenic leukocytes and other platelet-derived components such as platelet microparticles, serotonin, and leukocyte-associated mediators, promote endothelial cell proliferation, migration, and differentiation thus supporting neovascularization (317). This is complemented by modulation of endothelial permeability and vessel maturation and stabilization processes via angiopoietin-1/Tie2 signaling (318). Notably, an imbalance and dysfunction in this signaling pathway, which can be effectively modulated by platelet-rich derivatives, has been detected in rosacea lesions (319).

*In vivo* experiments have further demonstrated that platelet-rich derivatives significantly modulate key inflammatory pathways and improve disease symptoms in cutaneous and mucocutaneous disorders characterized by primary or secondary inflammation leading to tissue injury and disease progression. In this sense, *in vivo* preclinical evidence evaluating platelet-rich plasma (PRP) and related platelet-rich products in immune-mediated dermatologic disease models is largely restricted to the imiquimod (IMQ)-induced psoriasiform dermatitis model in mice and rats, with additional but more limited studies in chemically induced contact hypersensitivity models. Murine models of psoriasis have consistently demonstrated that platelet-rich derivatives exert robust anti-inflammatory and immunomodulatory effects. Both PRP and platelet lysate have been shown to significantly attenuate psoriasiform histopathological alterations, including hyperkeratosis, parakeratosis, acanthosis, and dermal inflammatory infiltrates. These morphological improvements were accompanied by a downregulation of key pro-inflammatory and proliferative mediators, including IL-17, NF-κB, and proliferating cell nuclear antigen (PCNA), reflecting a coordinated suppression of both inflammatory signaling and keratinocyte hyperproliferation (320). In particular, platelet-derived extracellular vesicles (PEVs) have been identified as the main bioactive component of PRP, mediating anti-psoriatic effects in IMQ-induced models through mitochondrial transfer and subsequent suppression of M1 macrophage polarization (321). *In vivo* evidence regarding the use of platelet derivatives in eczematous dermatitis remains limited and is primarily restricted to murine models of radiation-induced skin injury, which are commonly used as an experimental paradigm of cutaneous tissue damage and inflammatory response, indicating that PRP enhances K14 keratinocyte function via AKT signaling and accelerates the regeneration of irradiated skin (322).

#### Epithelial barrier restoration and mucocutaneous homeostasis

Epithelial barrier disruption is a central feature of many inflammatory dermatoses, characterized by impaired stratum corneum integrity and increased permeability, which result in elevated transepidermal water loss, dysregulated cutaneous immune responses and increased susceptibility to environmental or microbial triggers (29). In preclinical models, platelet-rich formulations restore mucocutaneous homeostasis via integrated regulation of epithelial structural integrity, hydration dynamics, and epithelial–immune crosstalk (38, 323, 324). Complementing canonical reparative signaling pathways, platelet-released mediators, including PDGF, TGF-β, EGF, together with plasma-derived factors such as IGF-1, converge on epithelial signaling networks, primarily involving EGFR, PI3K/Akt, and MAPK/ERK-dependent pathways, to reprogram differentiation trajectories required for barrier reconstitution (325, 326). This includes reinforcement of epidermal junctional complexes and restoration of stratum corneum structural proteins and lipid-associated cornified envelope components components in keratinized epithelia, resulting in the re-establishment of epithelial cohesion and regulation of paracellular and trans-epidermal permeability (38, 327, 328). Lipid barrier recovery is supported by PRP-mediated modulation of keratinocyte differentiation programs, which coordinate epidermal lipid organization required for extracellular lipid lamellae formation and restoration of barrier function (329). Concurrently, platelet-rich signaling may modulate inflammatory responses in wound microenvironments, contributing to a permissive environment for epidermal repair and barrier reconstitution (330). Collectively, these processes contribute to restoration of epithelial barrier function, thus regulating transepidermal water loss (TEWL) and epithelial hydration homeostasis (262).

#### Host microenvironment and microbiome interactions

Cutaneous and mucocutaneous health and disease is critically shaped by the dynamic interplay between microbial communities and the local tissue environment, where epithelial, immune and microbial components constitute an integrated regulatory network (331). In addition to infectious dermatoses, the role of microbiome has been highlighted in cutaneous autoiimnune conditions (332) and immune-mediated disorders (333, 334). The effects of platelet-rich formulations on host microenvironment–microbiome interactions are largely secondary to their primary role in restoring epidermal barrier homeostasis. By reinforcing epidermal structural integrity and normalizing barrier function, these preparations reduce microbial penetration and limit secondary dysbiosis-associated immune activation in barrier-compromised epithelial tissues (335). Limited evidence from *in vivo* studies suggest that platelet-rich derivatives may indirectly influence host–microbiome interactions. By reducing inflammation-induced dysbiosis and improving epithelial integrity, these formulations may contribute to stabilization of the cutaneous microenvironment, although disease-specific microbiome models remain limited. In this sense, a preliminary clinical study reported distinct ecological patterns in androgenetic alopecia patients treated with platelet derivatives compared with untreated patients. In this context, PRP administration has been associated with a compositional shift characterized by increased abundance of *Cutibacterium* and reduced proportion of *Staphylococcus*, potentially reflecting partial restoration of scalp microenvironment homeostasis (336).

Moreover, *in vitro* epithelial and microbial co-culture systems have consistently demonstrated that platelet derivatives exert antimicrobial activity, potentially related to their content of platelet-derived peptides and immune-modulatory mediators (337–342). This antimicrobial activity also targets multidrug resistant bacteria (343) and has been proposed as a potential prophylactic strategy in specific clinical contexts (344–346). In particular, antimicrobial effects associated with platelet derivatives have been identified against different microorganisms associated with dermatoses involving pronounced immune-mediated inflammation, including *Candida, Staphylococcus aureus* or *Cutibacterium acnes* (347–351). Their antimicrobial efficacy has been reported to be independent of leukocyte content (352, 353) but correlelated with the degree of platelet activation (354). Among *in vivo* models demonstrating concomitant reductions in inflammation, enhanced tissue repair, and decreased pathogen burden, those involving *Staphylococcus aureus* and *Candida albicans* are the most relevant to mucocutaneous pathology (355), (356). Nevertheless, its translational and clinical relevance remains to be fully established.

### Clinical evidence

Despite major advances in the mechanistic understanding of mucocutaneous immunopathology and the development of targeted therapies, current therapeutic strategies for managing dermatoses with a significant immune-inflammatory component remain largely suppressive rather than curative (11). Together with conventional systemic therapies, topical agents, and phototherapy, biologic agents and small-molecule inhibitors targeting key inflammatory pathways, particularly TNF-α, the IL-23/Th17 axis, and JAK/STAT signaling, have significantly improved disease control in conditions such as psoriasis and atopic dermatitis (357–361). However, their clinical effectiveness is constrained by heterogeneous and incomplete response rates, primary or secondary treatment failure, adverse effects and contraindications and the requirement for continuous long-term administration to maintain disease suppression (362–366). Furthermore, the redundancy and plasticity of mucocutaneous immune networks, involving overlapping innate, adaptive, neuroimmune, and microbiome-associated pathways, limit the efficacy of single-axis therapeutic targeting in many dermatoses (367). Additionally, high economic costs and global inequities limit access to advanced biologic and targeted therapies (368, 369). Collectively, these limitations underscore the need for long-term mechanism-based therapeutic approaches aimed at restoring immune homeostasis rather than transiently inhibiting discrete inflammatory mediators. In this context, autologous platelet-rich products have emerged as promising adjunctive or potentially disease-modifying therapeutic strategies due to their combined immunomodulatory and regenerative properties (38).

Detailed information on the synthesized clinical reports is provided in Supplementary Table S1. Clinical evidence regarding the use of platelet-rich derivatives in most cutaneous and mucocutaneous disorders in which dysregulated inflammatory or immune-related tissue response programs contribute to tissue injury and clinical phenotype is largely restricted to small studies, case series, and exploratory trials (232). In this context, psoriasis represents a developing target for PRP-based therapies. To date, a single randomized controlled trial (RCT) conducted by Mona et al. has been identified, concluding that PRP significantly reduced plaque size and thickness compared with saline at the 10-week follow-up. Furthermore, histopathological analysis of pre- and post-treatment biopsies demonstrated that PRP induced moderate to severe inhibition of IL-17 expression (370). Beyond this trial, the available clinical evidence is largely limited to preliminary studies. In a retrospective case series including 30 patients with psoriasis, Kauhl et al. (371) reported a significant reduction in average lesion size, and 80% of patients achieved complete remission after 12 weeks following subdermal PRP injections. These results are consistent with those reported by Chakravdhanula et al. in a prospective controlled pilot study, who observed that PRP combined with methotrexate (MTX) led to a substantial reduction in erythema, induration, and desquamation in patients with chronic plaque psoriasis when compared to MTX monotherapy. In their study, 62.5% of patients achieved a ≥75% improvement in the Psoriasis Area and Severity Index (PASI 75), while 12.5% reached PASI 90 after 16 weeks. Subsequent studies have corroborated these findings, also demonstrating a significant reduction in PASI scores exceeding 80% in patients treated with intralesional PRP combined with MTX at the 16-week follow-up (372). A second prospective pilot study conducted by Rahman et al. (373) reported a significant reduction in PASI scores and improved patient satisfaction in chronic, localized, recalcitrant psoriatic plaques treated with intralesional PRP, with clinical benefits emerging from week 8 onward and persisting through week 16. In a retrospective case series conducted by Anwar et al., the therapeutic potential of PRP for psoriasis was evaluated in 73 patients with psoriatic plaques who received intralesional PRP in combination with MTX. Treatment success was defined as achieving PASI 75 after 16 weeks. Based on this criterion, 82.19% of participants responded successfully to treatment, while the remaining 17.81% did not reach the required level of clinical improvement (374). However, the limited sample size and the lack of long-term follow-up data limits robust assessment of the durability and sustained clinical effectiveness of the observed therapeutic effects.

Published findings from preliminary clinical studies, small patient cohorts and case reports also suggest potential clinical benefits in atopic dermatitis, including reductions in inflammation, scaling, pruritus, itching and long-term symptom relief alongside subjective improvement in symptom burden (375–377). In the same line, Kauhl et al. observed a progressive reduction in lesion size, together with a consistent improvement in Eczema Area and Severity Index (EASI) scores in response to PRP therapy, with 50% of patients achieving complete clinical remission after 3 months (371). These results are consistent with those reported in a retrospective case series conducted by García-Millán et al., who concluded that intradermal PRGF administration improved skin hydration and reduced inflammatory infiltrate formation, thus attenuating skin inflammation and leading to enhanced clinical outcomes and higher patient satisfaction levels in patients with sensitive skin alterations, a condition frequently associated with atopic dermatitis (378). Notably, the highest level of clinical evidence currently available is provided by a split-body RCT, which demonstrated that PRP combined with narrowband ultraviolet B (NB-UVB) induced a significant improvement on EASI scores and a lower frequency of recurrence compared to the NB-UVB control group in patients with moderate to severe atopic dermatitis (379). In addition to atopic dermatitis, a growing number of preliminary clinical reports suggest that the potential therapeutic benefits of platelet-derived products may extend to other forms of dermatitis, steroid-induced (380) and sebhorreic dermatitis (381). In radiation-induced dermatitis, the evidence is particularly robust, with two RCTs demonstrating that platelet-rich therapies delayed the progression of acute radiation dermatitis or accelerated lesion healing, reduced pain, improved patient-reported outcomes, and reported no treatment-related adverse events (382, 383). These observations are further supported by evidence derived from case series and case reports (384–386).

Emerging clinical evidence supports the use of platelet-rich derivatives for treatting multiple inferface or lichenoid disorders. In this sense, the available clinical evidence is particularly robust in the case of lichen planus, with a total of 11 RCTs identified to date. Overall, the available data suggest that both PRP and PRF may represent potential alternatives to intralesional corticosteroids for the management of lichen planus (42, 387), with several studies reporting comparable or superior outcomes in terms of clinical improvement and pain reduction and minimal side-effects among groups receiving platelet derivatives (388–397). These conclusions are in agreement with preliminary findings reported across multiple prospective single-arm studies, cohort studies, case series and case reports (397–403). In the same line, data from retrospective studies concluded that PRGF may be considered a promising strategy for the treatment of erosive lichen planus refractory to steroid therapy (404, 405). It should be highlighted that the use of PRGF in the treatment of oral lichen planus represents one of the earliest systematically evaluated clinical approaches within the field of platelet-derived therapies for inflammatory mucocutaneous pathologies. In particular, a case series published by Piñas et al. (406) made a pioneering contribution to establishing the clinical basis for PRGF administration in refractory lichen planus, preceding the subsequent expansion of clinical investigations involving PRP and PRF for this indication. However, conflicting results have been reported in RCTs evaluating PRP-based platelet gel. According to Shalby et al., although a PRP-based platelet gel was associated with significant reductions in pain, lesion size, and erythema scores after 4 months of treatment in relation to baseline scores in patients with lichen planus, these reductions were not statistically significant compared with the placebo gel (396). Conversely, Huang and Li reported that PRP gel significantly reduced lesion area and pain after 4 weeks compared with Tacrolimus gel. These findings, together with an increase in CD3 and CD4-positive T lymphocytes (CD3+ and CD4+ T cells) and in the cluster of differentiation 4 to 8 ratio (CD4/CD8 ratio) compared with controls, may indicate modulation of T-cell subsets consistent with immune homeostasis restoration (407).

Other prospective single-arm pilot studies, case reports and case series have suggested its potential effectiveness and safety in the treatment of other related pathologies, including lichen planus pigmentosus (408) or lichen planopilaris (409–413). In the latter case, evidence from observational and exploratory studies is further supported by a RCT involving 24 patients with lichen planopilaris, which demonstrated that PRP significantly reduced the Lichen Planopilaris Activity Index (LPPAI) compared with corticosteroid therapy and was associated with higher patient-reported satisfaction at 6-month follow-up (414). Additionally, two retrospective studies have reported positive outcomes following PRP and PRGF administration in terms of hair loss, hair regrowth and scalp inflammation in the treatment of frontal fibrosing alopecia, considered a specific clinical variant of lichen planopilaris (415, 416). Regarding genital lichen sclerosus, the published evidence on PRP includes one placebo controlled RCT that failed to identify significant histopathological differences in inflammation between groups (417) and a randomized controlled pilot study PRP reduced vulvar inflammation and improved most clinical signs and symptoms of vulvar lichen sclerosus (418). Preliminary results from multiple single-arm studies also suggest that the administration of PRP and other platelet derivatives as monotherapy may contribute to the reduction or resolution of clinical manifestations associated with vulvar or penile lichen sclerosus (419–429). These findigs are consistent with those reported in certain isolated retrospective observational studies and case series (430, 431). Combination therapies based on PRP and other regenerative approaches such as adipose-derived stem cells (ADSCs), adipose-derived stromal vascular fraction (AD-SVF), and nanofat have also demonstrated that these interventions can attenuatte inflammation and improve most clinical signs of lichen sclerosus, particularly in patients refractory to conventional therapy (432). However, the evidence supporting these approaches remains limited, as it is primarily derived from retrospective studies and pilot trials (419, 433)

Other immune-mediated cutaneous pathologies include vitiligo and alopecia areata, which share overlapping autoimmune and immunogenetic susceptibility. In the case of vitiligo, clinical literature syntheses and latest evidence from RCTs consistenly converge in demonstrating significant improvements and repigmentation percentages following PRP administration, particularly in combination with CO_2_ or excimer laser therapy (434–441), but also when used as a monotherapy (442) or combined with other approaches (443). Lower-level clinical evidence, including prospective uncontrolled and self-controlled controlled studies, case series and case reports also provides support for PRP as a promising approach for the treatment of vitiligo (444–448). Findings from multiple RCTs also indicate that platelet-rich formulations improve hair regrowth while reducing alopecia severity scores, cell proliferation markers, hair dystrophy and/or burning or itching sensation in patients with alopecia areata, with efficacy and safety profile comparable to or exceeding that of intralesional corticosteroids or low level laser therapy (449–454). However, results from individual RCTs remain inconsistent, with PRP demonstrating limited clinical efficacy compared with placebo (455), intralesional corticosteroids (456–458) or combination therapies with fractional CO_2_ laser or microneedling (459). Positive outcomes derived from intradermal PRP administration in patients with alopecia areata have also been documented in several non-randomized trials, including hair regrowth or hair density (450, 460–462) or recurrence (463). Certain refractory cases of follicullitis decalvans, a rare subtype of chronic neutrophilic inflammatory cicatricial alopecia, have also demonstrated a symptomatic improvement in scalp inflammation following PRP interventions in combination with triamcinolone (464). A similar situation can be observed in hidradenitis suppurativa, where evidence for PRP is limited to preliminary studies and case reports describing favorable clinical outcomes, with no RCTs available (465–469). Regarding acne, the extensive available literature has been synthesized in multiple systematic reviews involving hundreds of patients from 34 individual primary studies, including 14 RCTs, 17 non-randomized split-face studies and 3 uncontrolled case series (470). The majority of identified studies have focused on the management of atrophic scarring using platelet-rich formulations either as a monotherapy (471–474) or in combination with microneedling (475, 476) and fraccional laser therapy (477–480), with reported significant improvements in the overall clinical appearance associated with PRP (481).

Among neurovascular inflammatory dermatoses, most clinical investigations have focused on rosacea, where preliminary reports limited to prospective split-face controlled studies and retrospective case series suggest that PRP-based therapies may be effective and safe therapeutical approaches to modulate inflammatory manifestations and reduce rosacea grading scores (380, 482–484). When considering infectious dermatoses characterized by clinically relevant inflammation, platelet-rich derivatives have been proposed as a potential adjunctive therapy, based primarily on experimental evidence. *In vitro* and *in vivo* studies suggest that bioactive molecules contained in these formulations may exert immunomodulatory, antimicrobial and regenerative effects. However, despite these biologically plausible mechanisms, clinical evidence remains scarce, with only limited clinical reports available. In this context, the most relevant clinical evidence derives from a meta-analysis involving a total of 7 RCTs, which concluded that pulsed radiofrequency combined with PRP is more effective that the traditional therapy based on pulsed radiofrequency for herpes zoster-associated neuralgia (485). Moreover, a systematic synthesis of 17 RCTs, five single-arm studies, two retrospective studies and one case report that indicates that PRP alone or combined with other therapies improved pain control and sleep quality and reduced the incidence of postherpetic neuralgia and analgesic use in patients with herpes zoster (486). A comparable scenario has also been observed in relation to SCARSs, for which evidence remains extremely limited due to their rarity and high clinical severity, although preliminary findings appear promising. In this sense, Klama-Baryla et al. (487) reported a case in which biostatic human amnion and platelet-rich plasma exerted positive effects in the management of toxic epidermal necrolysis.

In contrast, the use of platelet-rich derivatives in autoimmune cutaneous and mucocutaneous disorders has received substantially less research focus, with available evidence consisting primarily of prospective pilot studies, case series and case reports. Platelet derivatives have been proposed as valid approaches to treat morphea (488, 489), resistant oral erosions of pemphigus vulgaris (490–494) and pemphigus foliaceous wounds (495). In particular, a split-mouth randomized double blind pilot study study conducted by El-Komy et al. in patients with pemphigous vulgaris observed significant improvements in clinical outcomes, Pemphigus Disease Area Index (PDAI), and pain scores at 3-month follow-up, with no statistically significant differences between PRP and corticosteroid injection sites (496). Isolated reports also suggest that adjunt PRP-based therapies may be regarded as a viable option for managing mucocutaneous manifestations of lupus erythematosus, particularly for stabilizing the progression on inflammatory cicatricial alopecia (497–499). Similar conclusions can be drawn for certain immuno-mediated chronic disorders with a potential neurovascular component. For example, Huber et al. (255) highlighted that PRP administration in patients with Behçet's disease induced an anti-inflammatory profile characterized by increased Treg cells and decreased activated NK cells and alterations in cytokine profiles. Moreover, a clinical improvement was observed as revealed by a decrease in the number and time of closure of oral ulcers (255). In addition, recent clinical systematic reviews suggest that platelet-rich plasma may improve neuropathic pain, potentially via modulation of the inflammatory microenvironment and tissue repair processes. This is particularly important since, in chronic inflammatory dermatoses, persistent neuroimmune interactions may lead to peripheral nerve sensitization and dysregulation, thus contributing to neuropathic pain–associated symptoms (500). Table 1 summarizes the current clinical evidence and the main conclusions for each disease indication.

**Table 1:** Summary of the clinical evidence identified.

Disease	Highest level of clinical evidence	Main evidence base	Main limitations	Overall level of clinical evidence
Psoriasis	RCT (370)	Prospective controlled pilot studies and retrospective case series (371–374)	Most evidence derived from preliminary studies; small sample size; short follow-up periods; methodological heterogeneity	Emerging evidence
Atopic dermatitis	RCT (379)	Retrospective case series and reports (371, 375–378)		
Dermatitis (radiation, steroid-induced and sebhorreic dermatitis)	RCT (382, 383)	Retrospective case series and reports (380, 381, 384–386)	Most evidence derived from preliminary studies; small sample size; short follow-up periods; methodological heterogeneity; clinical heterogeneity	Emerging evidence in radiation-induced dermatitis and preliminary evidence in steroid-induced and sebhorreic dermatitis
Lichen planus	Systematic review of RCTs (42, 387)	RCT (388–397, 407, 414), prospective single-arm pilot study, retrospective cohort studies, case series and reports (398–406, 408, 410–412, 415, 416, 419, 420, 431)	Methodological heterogeneity	Growing evidence
Lichen sclerosus	RCT (417, 418)	Prospective self-controlled studies, prospective single-arm pilot studies, retrospective cohort studies, case series and reports (418, 421–428, 430, 432, 433)	Most evidence derived from preliminary studies; small sample size; short follow-up periods; methodological heterogeneity; inconsistent findings	Preliminary evidence
Vitiligo	Meta-analysis from RCT (434, 435, 437–439)	RCTs, prospective self-controlled studies, prospective single-arm pilot studies, retrospective cohort studies, case series and reports (436, 440, 442–448)	Methodological heterogeneity; combination therapies predominate	Growing evidence
Alopecia areata	Meta-analysis from RCT (449)	Retrospective cohort studies, case series and reports (453, 458–461, 463)	Methodological heterogeneity; inconsistent findings	Growing evidence
Inflammatory follicular disorders (folliculitis decalvans and hidradenitis suppurativa)	Retrospective comparative study (465)	Retrospective cohorts, case series and reports (464, 466, 467, 469)	Most evidence derived from preliminary studies; small sample size; short follow-up; methodological heterogeneity	Preliminary evidence
Acne	Overview of systematic reviews including RCTs and non-randomized interventional studies (470)	RCTs, prospective self-controlled studies, prospective single-arm pilot studies, retrospective cohort studies, case series and reports (471–474, 479, 480)	Methodological heterogeneity; combination therapies predominate; focus mainly on acne scars	Growing evidence
Rosacea	RCT (482)	Retrospective case series (380, 483, 484)	Most evidence derived from preliminary studies; small sample size; short follow-up; methodological heterogeneity	Preliminary evidence
Infectious dermatoses	Meta-analysis from RCTs (485)	Synthesis of RCTs, prospective single-arm pilot studies, retrospective case series and reports (486, 524)	Methodological heterogeneity; combination therapies predominate; focus mainly on zoster associated neuropathic pain	Growing evidence
SCARs	Case report (487)	None	The application is supported only by anecdotal evidence from a single case report	Anecdotal evidence
AIBDs	Pilot RCT (496)	Prospective single-arm pilot studies, retrospective case series and reports (488–495, 497–499)	Most evidence derived from preliminary studies; small sample size; short follow-up; methodological heterogeneity	Preliminary evidence

### Standardization and minimum reporting requirements

The inconsistent adherence to minimum reporting standards and essential characterization requirements in terms of platelet enrichment, leukocyte content or activation status across studies significantly compromises the comparison and synthesis of the available clinical evidence on platelet-rich formulations (501). This limitation is particularly relevant considering that the regenerative and immunomodulatory properties of platelet-rich derivatives are highly dependent on multiple processing variables, including anticoagulant use, centrifugation protocol (rotor design, speed, duration, and number of cycles), fractionation method and the presence or absence of exogenous activation (502). Additional sources of variability associated with the use of platelet-rich derivatives, such as storage conditions, timing of processing and administration, delivery route and dosing regimen, further contribute to substantial heterogeneity in the final product composition and local bioavailability (503). Despite the fact that there is currently no consensus regarding the optimal formulation or preparation protocol for platelet-rich derivatives across different clinical indications, consistent implementation of minimal standardized reporting of compositional and methodological parameters would substantially enhance reproducibility and enable a more accurate identification of the key factors driving clinical response (504). This approach would further support the correlation between specific compositional profiles and clinical outcomes, thus enhancing the interpretability of existing studies and guiding the optimization of future applications (34).

## Discussion

A substantial body of clinical evidence supports the use of platelet-rich products across a range of dermatosis involving primary or secondary inflamatory mechanisms associated with tissue injury and disease progression. In particular, consistent positive outcomes have been reported in psoriasis, atopic dermatitis, multiple clinical variants of lichen planus and lichen sclerosus, vitiligo, alopecia areata, and acne, where improvements in symptom severity, tissue inflammation markers and clinically assessed or patient-reported outcomes have been frequently observed. In several studies, platelet-rich therapies have demonstrated outcomes comparable to, or in some cases exceeding, those observed with conventional therapies. Complementary to this, encouraging results have been described in other clinically relevant conditions, including rosacea and hidradenitis suppurativa, although the level of evidence remains more limited in these pathologies. Moreover, isolated clinical reports and preliminary studies suggest potential benefits in selected autoimmune mucocutaneous disorders, infectious dermatoses associated with a significant inflammatory component and SCARs, among other conditions, highlighting the broad but still evolving therapeutic target of interventions based on platelet-rich derivatives.

Although ocular surface disorders, including dry eye disease and autoimmune-related epithelial surface diseases, share relevant pathogenic mechanisms with cutaneous and mucocutaneous inflammatory conditions, including epithelial barrier dysfunction, immune activation, and impaired tissue repair, these disorders were not included in the present review. This decision was based on the distinct anatomical and physiological characteristics of the ocular surface, together with the extensive and rapidly expanding literature specifically addressing ophthalmic applications of platelet-derived therapies, which would have substantially broadened the scope and clinical heterogeneity of the present analysis (505–508). Nevertheless, emerging evidence from ocular surface research provides valuable complementary insights into the regenerative and immunomodulatory potential of platelet-rich formulations in epithelial barrier disorders (509–513).

Overall, the observed clinical effects of platelet-rich derivatives can be broadly interpreted through five interconnected biological processes: immune modulation, epithelial barrier restoration, angiogenesis and tissue repair, extracellular matrix remodeling, and normalization of epithelial–immune–microbiome interactions. These coordinated mechanisms are mediated by the release of growth factors, cytokines, chemokines, extracellular vesicles, and other bioactive molecules, which collectively contribute to the modulation of inflammatory pathways, promote tissue regeneration processes, and restore local tissue homeostasis. Platelet-rich therapies represent the most clinically established autologous biologic approach in dermatology, combining a favorable safety profile, low mufacturing complexity and broad clinical availability. In contrast, other regenerative strategies, including mesenchymal stromal cell-derived secretome, extracellular vesicles (exosomes), and cell-based therapies, remain largely experimental for most dermatologic indications despite promising preclinical and early clinical results. While these emerging biologics may provide more targeted immunomodulatory and regenerative effects, they face greater challenges regarding standardization, regulatory approval, and clinical translation (514, 515). Consequently, platelet-rich therapies currently occupy a unique translational position, providing the most accessible and extensively investigated autologous regenerative platform for routine dermatological practice.

Other regenerative biologics, particularly placental and perinatal-derived products, have emerged as promising therapeutic approaches for inflammatory and regenerative dermatology. Similar to platelet-rich formulations, these products exert multimodal immunomodulatory and regenerative effects through the delivery of bioactive mediators. However, they differ substantially in their biological source, composition, manufacturing processes, and regulatory considerations. In this sense, placental-derived therapies may provide additional extracellular matrix components, extracellular vesicles, and tissue-specific bioactive molecules, whereas platelet-rich derivatives represent autologous formulations characterized by a more favorable safety profile and broader clinical experience. In contrast, placental and perinatal-derived products are allogeneic biologics that require tissue procurement, donor screening, specialized processing and storage procedures, and more complex regulatory oversight, which may limit their accessibility and widespread clinical implementation. Despite these differences, both approaches currently face challenges related to product standardization, methodological heterogeneity, and the need for adequately powered comparative clinical trials before their relative therapeutic value can be established (516, 517). At present, placental and perinatal-derived products should be considered complementary rather than competing regenerative strategies, as there is insufficient comparative evidence to establish the superiority of either approach or to define their respective indications in inflammatory skin diseases and regenerative dermatology.

As evidenced from the present review, the limited quality of most available clinical findings and the substantial heterogeneity identified among pathological conditions, study designs, formulations evaluated, administration protocols, outcome measures, and study populations, together with the limited availability of large-scale controlled trials, currently restricts the interpretability, reproducibility, and broader applicability of the available evidence. Importantly, differences in platelet-rich formulation protocols, including platelet and leukocyte content, anticoagulant use, centrifugation methods, activation strategies, treatment regimens, and administration techniques, represent additional sources of variability that may substantially influence clinical outcomes and limit direct comparisons across studies (34, 518–521). This methodological variability directly determine the biological composition and functional properties of platelet-derived products, potentially resulting in clinically relevant differences in therapeutic effects and contributing to the heterogeneous outcomes reported across clinical studies (449, 522, 523). Future clinical studies should adhere to standardized minimum reporting criteria for platelet-rich derivatives to minimize confounding factors associated with the composition, manufacturing, and administration of platelet-rich products, as these variables may critically influence the clinical efficacy of these autologous therapies. In this context, the implementation of minimal reporting standards would facilitate between-study comparability and facilitate the identification of key determinants of therapeutic response.

Despite the growing body of preclinical and clinical evidence supporting the potential role of platelet-rich formulations across a wide spectrum of cutaneous and mucocutaneous disorders involving a significant role of primary or secondary inflammation leading to tissue injury and clinical phenotype, the current literature is characterized by important methodological limitations that preclude definitive clinical recommendations. Most available reports are based in studies limited by small sample sizes, short follow-up periods, heterogeneity in study design and a predominance of non-controlled or non-randomized designs, which collectively increase the risk of bias and limit reproducibility. A particularly critical aspect is the composition and administration regimens of the platelet-rich products employed, which can exert a substantial impact on clinical outcomes and may contribute significantly to the heterogeneity observed across studies. In addition, outcome measures are often inconsistent across studies, frequently relying on subjective or semi-quantitative clinical scores rather than standardized immunological or histopathological endpoints. The inherent variability across study parameters and experimental designs highlights the need for detailed and standardized reporting of methodology and results.

Moreover, the variability among study populations and the absence of large-scale, multicenter randomized controlled trials for many indications, together with a lack of head-to-head comparisons against established standard-of-care therapies, significantly constrains the ability to determine the true magnitude and long-term efficacy of these formulations. The predominance of short follow-up periods also restricts the robustness of conclusions regarding long-term effectiveness and safety outcomes. Limited interstudy comparability also persists resulting fom insufficient adherence to methodological standards and reporting principles regarding platelet-rich composition and dosing regimens. Consequently, although treatments based on platelet-rich preparations constitute a biologically plausible, multimodal regenerative approach capable of modulating immune-responses and supporting tissue repair, there is currently no consensus regarding their role as disease-modifying therapies and they are generally considered promising adjunctive interventions. Future research should prioritize standardized formulation protocols, mechanistically driven endpoints, and properly designed controlled trials with long-term follow-up periods. Disease-specific characteristics and patient subgroups associated with a greater therapeutic response should also be identified to better define which patients may benefit from platelet-rich therapies and how these interventions can be optimally integrated into evidence-based management strategiesfor dermatoses associated with a significant immune-inflammatory component.

Additionally, the substantial heterogeneity of the diseases included in this review, involving multiple etiopathogenic mechanisms and varying molecular and immunoinflammatory pathways, together with the multifactorial nature of many of these conditions, represents a major limitation in the interpretation of the overall safety and efficacy of interventions based on platelet-rich derivatives. This heterogeneity reflects a well-established clinicopathological dissociation, in which different etiological triggers may converge on common immunopathological pathways, while similar clinical phenotypes may result from heterogeneous underlying pathogenic mechanisms. Furthermore, variability in diagnostic criteria and disease classification systems across studies further increases methodological inconsistency and reduces inter-study comparability. Collectively, this biological, methodological and reporting-related heterogeneity substantially limits the interpretability and generalizability of pooled outcomes associated with therapies involving platelet-rich derivatives.

## Glossary

AD-SVF, Adipose-derived stromal vascular fraction; ADP, Adenosine diphosphate; ADSCs, Adipose-derived stem cells; AIBD, Autoimmune blistering diseases; APC, Antigen-presenting cell(s); CCL, CC chemokine ligands; CD8+ T cells, Cytotoxic T lymphocytes; CGRP, Calcitonin gene-related peptide; c-MET, HGF receptor (MET proto-oncogene receptor tyrosine kinase); CXCL, CXC chemokine ligands; CXCL9/CXCL10, Interferon-inducible chemokines; CXCL12, Stromal cell-derived factor 1; CXCR3, CXC chemokine receptor 3; DAMPs, Damage-associated molecular patterns; EGF, Epidermal growth factor; EGFR, Epidermal growth factor receptor; EASI, Eczema Area and Severity Index; FGF-2, Fibroblast growth factor 2; FOXP3, Forkhead box P3; HGF, Hepatocyte growth factor; IFN-γ, Interferon gamma; IGF-1, Insulin-like growth factor 1; IL-1, Interleukin-1; IL-4, Interleukin-4; IL-5, Interleukin-5; IL-6, Interleukin-6; IL-10, Interleukin-10; IL-13, Interleukin-13; IL-17, Interleukin-17; IL-23, Interleukin-23; IL-31, Interleukin-31; JAK/STAT, Janus kinase/signal transducer and activator of transcription pathway; K14, Keratin 14; LL-37, Cathelicidin antimicrobial peptide; LPPAI, Lichen Planopilaris Activity Index; MAPK, Mitogen-activated protein kinase pathway; MHC, Major histocompatibility complex; MMP, Matrix metalloproteinases; M2 macrophage, Alternatively activated macrophage phenotype; MALAT1, Metastasis-associated lung adenocarcinoma transcript 1; NF-κB, Nuclear factor kappa-light-chain-enhancer of activated B cells; NK cells, Natural killer cells; NLR, NOD-like receptors; PAMPs, Pathogen-associated molecular patterns; PASI, Psoriasis Area and Severity Index; PDAI, Pemphigus Disease Area Index; PDGF, Platelet-derived growth factor; PEVs, Platelet-derived extracellular vesicles; PI3K/AKT, Phosphoinositide 3-kinase/protein kinase B signaling pathway; PRGF, Plasma rich in growth factors; PRP, Platelet-rich plasma; PRR, Pattern recognition receptors; RLR, RIG-I-like receptors; ROS, Reactive oxygen species; SCAR, Severe cutaneous adverse reactions; SIRT1, Sirtuin 1; SP, Substance P; STAT5, Signal transducer and activator of transcription 5; TEN, Toxic epidermal necrolysis; TEWL, Transepidermal water loss; TLR, Toll-like receptors; TNF-α, Tumor necrosis factor alpha; TGF-β, Transforming growth factor beta; TIMP, Tissue inhibitors of metalloproteinases; TRPV1, Transient receptor potential vanilloid 1; Th1, T helper 1 cells; Th2, T helper 2 cells; Th17, T helper 17 cells; Treg, Regulatory T cells; VEGF, Vascular endothelial growth factor; YAP/TAZ, Yes-associated protein/transcriptional coactivator with PDZ-binding motif.
