# Supplementary material for: Platelet-rich therapies in cutaneous and mucocutaneous disorders characterized by inflammatory tissue responses: mechanistic insights and clinical evidence
Source: Front Med (Lausanne). 2026 Sep 18;13:1901254. doi: 10.3389/fmed.2026.1901254 (PMC13631438; doi:10.3389/fmed.2026.1901254)
Supplement: Supplementary file 1 [file Supplementary_file_1.docx]

**Note S1.** Literature search strategy

A literature search was conducted in PubMed, Scopus, and Google Scholar to identify publications relevant to the immunomodulatory, regenerative, and reparative effects of platelet-rich derivatives in cutaneous and mucocutaneous disorders characterized by inflammatory tissue responses. The search strategy combined terms related to platelet-rich formulations (e.g. “PRP”,“platelet-rich plasma”, “PRGF”, “plasma rich in growth factors”, “PRF”, “platelet-rich fibrin”, “CGF”, “concentrated growth factor”, “platelet concentrates” or “platelet-derived products”) with terms related to inflammatory and immune-mediated skin and mucosal diseases (e.g. “psoriasis”, “dermatitis”, “lichen planus”, “lichen sclerosus”, “vitiligo”, “alopecia areata”, “acne”, “hidradenitis suppurativa”, “rosacea”, “AIBD” or “SCAR”) and their associated biological processes (e.g. “inflammation”, “immunomodulation”, “epithelial barrier”, “wound healing”, “regeneration”, “angiogenesis” or “tissue repair”). Boolean operators “AND” and “OR” were used to combine search terms and broaden or narrow the results as appropriate. Additional relevant publications were identified by manually screening the reference lists of retrieved articles and relevant review papers. Since this was a narrative review, publications were selected based on their relevance to the scope and objectives of the review, prioritizing original studies providing experimental or clinical evidence on the biological effects, mechanisms of action, and therapeutic applications of platelet-rich derivatives in cutaneous and mucocutaneous disease. Studies addressing related platelet-derived formulations were also considered when they provided relevant evidence for contextualizing the mechanisms or clinical potential of these therapies. No formal systematic-review or meta-analytic procedures were applied.
