# Supplementary material for: Platelet-rich therapies in cutaneous and mucocutaneous disorders characterized by inflammatory tissue responses: mechanistic insights and clinical evidence
Source: Front Med (Lausanne). 2026 Sep 18;13:1901254. doi: 10.3389/fmed.2026.1901254 (PMC13631438; doi:10.3389/fmed.2026.1901254)
Supplement: Supplementary file 2 [file Table_1.docx]

**Table S1.** Characteristics of the included studies

| **Condition** | **Study** | **Design** | **Study population (n)** | **Platelet-rich therapy** | **Comparator** | **Treatmen regimen** | **Follow-up** | **Main outcomes** | **Main findings** | **Level of evidence*** |
| --- | --- | --- | --- | --- | --- | --- | --- | --- | --- | --- |
| Psoriasis | Mona et al. (377) | RCT | 24 | PRP (24) | PBO (24) | Weekly (intradermal) | 10 weeks | Clinical evaluation, histopathological parameters and IL-17 expression | Psoriatic plaques treated with PRP showed statistically significant improvements considering clinical, histopathological and IL-17 expression compared to lesions with PBO. However, its therapeutic effect was mild | II |
| Psoriasis and atopic dermatitis | Kauhl et al. (378) | Retrospective case series | 40 | PRP | None | 3-weeks intervals (intradermal) | 12 weeks | Clinical evaluation: Psoriasis Area and Severity Index (PASI) and Eczema Area and Severity Index (EASI) | The average lesion size decreased significantly and 50 (dermatitis)-80% (psoriasis) patients achieved total remission | IV |
| Psoriasis | Chakravdhanula et al. (379) | Prospective controlled pilot study | 21 | PRP + MTX (16) | MTX (5) | Single ene (intralesional) + folitrax-15 (4 weeks) | 16 weeks | Clinical evaluation (PASI) and adverse events | Combination therapy with PRP + MTX significantly improved clinical signs of psoriasis and was well tolerated, with no serious adverse events | III |
| Psoriasis | Rahman et al. (380) | Prospective controlled pilot study | 30 | PRP (20) | Emollient (10) | Single ene (intralesional) | 16 weeks | Clinical evaluation (PASI) and physician/patient’s satisfaction | PRP demonstrated clinical enefit in patients with chronic localized recalcitrant plaque psoriasis and was well tolerated | III |
| Psoriasis | Anwar et al. (381) | Retrospective case series | 73 | PRP + methotrexate (MTX) | None | PRP three doses (1, 6 and 11 weeks) + MTX weekly (4 weeks) (intralesional) | 16 weeks | Clinical evaluation (EASI ) | PRP combined with MTX significantly improved psoriasis severity as reflected by a marked reduction in PASI score | IV |
| Atopic dermatitis | Ghani et al. (382) | Prospective case series | 2 | PRP | None | Three doses (15-days intervals) | 4 weeks | Biomarkers, clinical evaluation and patient’s satisfaction | PRP improved clinical manifestations of atopic dermatitis and demonstrated a favorable safety profile | IV |

*Continued on the next page*

***Table S1.*** *Continued*

| Atopic dermatitis | Karina et al. (383) | Retrospective case report | 1 | PRP | None | 15 doses (2-3-weeks interval) | Throughout pregnancy until delivery (maternal and fetal outcomes monitored until birth) | Clinical evaluation | Activated autologous PRP improved atopic dermatitis symptoms during pregnancy, with no reported maternal or fetal adverse effects. | IV |
| --- | --- | --- | --- | --- | --- | --- | --- | --- | --- | --- |
| Atopic dermatitis | Vafaei-Nodeh et al. (384) | Retrospective case report | 1 | PRP | None | Two doses (6-weeks intervals) | 1 year | Clinical evaluation and patient’s satisfaction | PRP was associated with sustained symptom improvement for up to 2 years, supporting its potential as a therapeutic option for atopic dermatitis. | IV |
| Atopic dermatitis | García-Millán et al. (385) | Retrospective case series | 5 | PRGF | None | Daily for 3 months (topical) | 3 months | Clinical assessment, patient’s satisfaction, skin hydration measurement and high-resolution topographic and reflectance confocal imaging analysis | Preliminary findings suggest that topical PRGF may improve clinical symptoms associated with sensitive skin | IV |
| Atopic dermatitis | Yosef et al. (386) | RCT | 44 | Narrowband-ultraviolet B (NB-UVB) + PRP (44) | NB-UVB (44) | 3-weeks intervals for 3 months (intradermal) | 6 months | Clinical evaluation (EASI ) | PRP combined with NB-UVB improved clinical outcomes and reduced treatment duration compared with NB-UVB alone | IV |
| Atopic dermatitis | Konuş et al. (387) | Retrospective case report | 1 | PRP | None | Two intradermal doses (4-weeks intervals) | 3 months | Clinical evaluation, patient’s satisfaction and adverse effects | PRP was associated with significant clinical improvement in steroid-induced rosacea-like dermatitis | IV |
| Atopic dermatitis | Álvarez et al. (388) | Retrospective case series | 20 | PRP + ketoconazol | None | Three doses (1-week intervals) | 3 months | Clinical evaluation | PRP was a safe and effective adjunctive treatment for seborrheic dermatitis, resulting in significant clinical improvement | IV |

*Continued on the next page*

***Table S1.*** *Continued*

| Radiation-induced dermatitis | Tao et al. (389) | RCT | 40 | Platelet-rich gel (APGR) (20) | vitamin B12 (20) | APGR sprayered and covered for 2-3 days and B12 in a wet compress for 30 min 3 times daily | 1 week | Clinical evaluation, patient’s satisfaction and adverse effects | APRG significantly accelerated wound healing, reduced pain, and improved quality of life compared with vitamin B12, with a favorable safety profile | II |
| --- | --- | --- | --- | --- | --- | --- | --- | --- | --- | --- |
| Radiation-induced dermatitis | Chen et al. (390) | RCT | 100 | Xenogeneic PRP lotion (52) | XONRID® gel (48) | Topical application throughout radiotherapy (twice a day) | 2 weeks post-radiotherapy | Clinical evaluation and patient’s satisfaction (RTOG dermatitis grade, NRS pain score, DLQI) | Xenogeneic PRP lotion reduced the severity and delayed the progression of acute radiation dermatitis, decreased pain, and improved quality of life compared with XONRID® gel | II |
| Radiation-induced dermatitis | Piccin et al. (391) | Retrospective case report | 1 | Platelet gel + hyperbaric oxygen | None | Topical application, 2–3 weeks, then every 2 weeks (40 applications total), combined with hyperbaric oxygen therapy (50 sessions) | 10 months | Clinical assessment | Combined platelet gel and hyperbaric oxygen therapy achieved complete wound healing and tissue regeneration in severe radiation dermatitis | IV |
| Radiation-induced dermatitis | Guberti et al. (392) | Retrospective case report | 1 | Homologous platelet gel | None | Twice daily (topical) on radiotherapy-free days (4 applications total) | 2 weeks | Clinical evaluation and patient’s satisfaction (RTOG grade, CTCAE v4.03, NRS pain, wound healing/reepithelialization | Homologous platelet gel promoted complete reepithelialization, reduced pain, and improved radiation dermatitis despite ongoing radiotherapy and cetuximab treatment | IV |
| Radiation-induced dermatitis | Iervolino et al. (393) | Prospective case series | 10 | Autologous platelet gel | None | Once weekly (topical) for a mean of 35 days | 5 years | Clinical evaluation (Wound healing, CTCAE grade, EPUAP classification) | Autologous platelet gel promoted healing in most patients with chronic radiation dermatitis refractory to conventional therapy, with sustained long-term lesion resolution in successfully treated cases | IV |

*Continued on the next page*

***Table S1.*** *Continued*

| Lichen planus | Niemczyk et al. (43) | Systematic review | 7 RCTs involving 122 patients | PRP + iPRF | Mainly intralesional corticosteroids | Variable | Variable | Clinical evaluation, patient’s satisfaction and adverse events | PRP and i-PRF improved pain and lesion severity, with efficacy generally comparable to corticosteroids in most RCTs. Further high-quality studies with standardized protocols are needed to confirm the role of platelet-rich concentrates as alternative or adjunctive therapies for the management of oral lichen planus | I |
| --- | --- | --- | --- | --- | --- | --- | --- | --- | --- | --- |
| Lichen planus | Ślebioda et al. (394) | Comprehensive review (systematic literature search) | 9 RCTs and 5 cohort studies, case-control and case reports involving 282 patients | iPRP, iPRF and platelet gel | Mainly intralesional corticosteroids, placebo gel or conventional therapy | Variable | Variable | Clinical evaluation, patient’s satisfaction and adverse events | Current evidence suggests that platelet-rich concentrates are promising, safe adjunctive treatments for lichen planus that may improve clinical outcomes, but larger, well-designed randomized controlled trials with standardized protocols are needed to confirm their efficacy | II |
| Lichen planus | Anitua et al. (414) | Retrospective case series | 3 | PRGF | None | Weekly perilesional infiltration of PRGF (1-3) | 6 months | Clinical evaluation and patient’s satisfaction (VAS, healing and recurrences), and adverse events | All patients showed marked symptom relief and lesion improvement without adverse events, suggesting PRGF as a promising alternative for oral lichen planus | IV |
| Lichen planus | Piñas et al. (412) | Prospective case series | 10 | PRGF | None | Weekly perilesional infiltration of PRGF (1-3) | 6 months | Clinical evaluation and patient’s satisfaction (VAS, healing and recurrences), and adverse events | PRGF significantly reduced pain and lesion size and promoted healing in patients refractory to corticosteroids, with no relevant adverse effects | IV |
| Lichen planus | Piñas et al. (413) | Retrospective case series | 10 | PRGF | None | Weekly perilesional infiltration of PRGF (1-3) | 6 months | Clinical improvement, histopathological changes and inflammation | PRGF improved clinical symptoms and reduced inflammatory infiltrate, supporting its regenerative and anti-inflammatory effects | IV |

*Continued on the next page*

***Table S1.*** *Continued*

| Lichen planopilaris | Kiatsurayanon et al. (416) | Prospective single-arm pilot study | 12 | PRP | None | Three doses at 2-week intervals (intradermal) | 12 weeks | Clinical evaluation, patient’s satisfaction and adverse events (PGA, PtGA), mMASI and VISIA® skin analysis) | PRP significantly improved hyperpigmentation and patient/physician assessments, with no serious adverse events | IV |
| --- | --- | --- | --- | --- | --- | --- | --- | --- | --- | --- |
| Lichen planopilaris | Bolanča et al. (417) | Retrospective case report | 1 | PRP | None | Three doses at monthly intervals (intradermal) | 6 months | Clinical improvement (trichometric measurements and photography) and histopathological evaluation | After PRP administration, the patient experienced complete regression of itching and shedding | IV |
| Lichen planopilaris | Svigos et al. (418) | Retrospective case series | 10 | PRP | None | Not reported | 3 months | Clinical improvement (trichometric measurements and photography) and inflammation | PRP was well tolerated without Koebnerization, and 4/10 patients experienced clinical improvement | IV |
| Lichen planopilaris | Jha(419) | Retrospective case report | 1 | PRP + Minoxidil | None | Four doses at 3-weeks intervals (intradermal) | 3 months | Clinical improvement | PRP may represent a valuable adjunct to topical minoxidil, with evidence suggesting enhanced hair thickening compared with topical minoxidil alone | IV |
| Lichen planopilaris | Osipowicz et al. (420) | Retrospective case series | 5 | PRP + FUE | None | FUE + PRP mesotherapy administered immediately after surgery to the donor and recipient areas, followed by three additional PRP mesotherapy sessions at 30-day intervals and one final PRP session 6 months after FUE | 12 months | Hair transplantation outcomes | Hair transplantation was successfully performed in selected patients with stable LPP | IV |

*Continued on the next page*

***Table S1.*** *Continued*

| Lichen planopilaris | Saxena et al. (421) | Retrospective case report | 1 | PRP + FUE + MN | None | Test patch FUE with scalp grafts + PRP injected into the recipient area. After successful engraftment, definitive FUE using scalp and beard grafts with an additional PRP injection into all recipient sites | 10 months | Hair transplantation outcomes | Successful hair restoration following combined hair transplantation and PRP | IV |
| --- | --- | --- | --- | --- | --- | --- | --- | --- | --- | --- |
| Lichen planopilaris | Behrangi et al. (422) | RCT | 24 | PRP + Clobetasol (12) | Clobetasol (12) | Three doses at monthly intervals (intradermal) | 6 months | Clinical assessment (LPPAI), physician’s and patient’s satisfaction, tolerability and adverse effects | PRP + clobetasol achieved greater and more sustained reductions in LPPAI and higher patient’s satisfaction than clobetasol alone, with good tolerability | II |
| Lichen planopilaris | Özcan et al. (423) | Retrospective case report | 1 | PRP | None | Five doses at monthly intervals (intradermal) | 5 months | Clinical and trichometric assessment of perifollicular erythema and scaling | Clinical improvement was reported in treatment-resistant frontal fibrosing alopecia after PRP therapy | IV |
| Lichen planopilaris | García et al. (424) | Retrospective comparative cohort study | 118 | PRGF + conventional therapy (61) | Conventional therapy (57) | PRGF injections every 2 to 4 months (intradermal) | 2 years | Clinical assessment (FFASS, alopecia extent and inflammation) | PRGF used as an adjuvant to standard therapy was associated with clinical improvement | III |
| Lichen sclerosus | Goldstein et al. (425) | RCT | 30 | PRP (20) | Saline (10) | Two PRP infiltrations (6-weeks intervals) | 12 weeks | Histopathologic inflammation, clinical assessment (CSS) and adverse events | PRP was not superior to placebo in improving histopathological inflammation or clinical symptoms | II |
|  |  |  |  |  |  |  |  |  |  |  |

*Continued on the next page*

***Table S1.*** *Continued*

| Lichen sclerosus | Gutiérrez-Ontalvilla et al. (426) | RCT | 20 | Nanofat + PRP (10) | Corticosteroids (10) | Two infiltrations (3-months interval) | 1 year | Vulvar skin elasticity, clinical assessment and patient’s satisfaction (clinical signs and symptoms) and histopathological assessment | Compared with topical corticosteroids, PRP reduced vulvar inflammation and improved most clinical signs and symptoms of vulvar lichen sclerosus, although no improvement in vulvar skin elasticity was observed | II |
| --- | --- | --- | --- | --- | --- | --- | --- | --- | --- | --- |
|  |  |  |  |  |  |  |  |  |  |  |
| Lichen sclerosus | Casabona et al. (427) | Prospective case series | 45 | PRP | None | Three PRP infiltrations (15-day intervals) | 12 months | Clinical assessment (IGA and DLQI) | PRP treatment for penile lichen sclerosus shows promise in reducing scarring, relieving symptoms, and improving quality of life, although further studies are needed to confirm its long-term efficacy | IV |
|  |  |  |  |  |  |  |  |  |  |  |
| Lichen sclerosus | Behnia-Willison et al. (428) | Retrospective case series | 28 | PRP | None | Three PRP infiltrations (4 to 6-weeks intervals) and at 12 months | 12 months | Clinical assessment, steroid use and patient’s satisfaction | PRP reduced lesion size and symptoms, with complete lesion resolution in 8 of 28 patients and elimination of the need for corticosteroid therapy in most cases |  |
| Lichen sclerosus | Goldstein et al. (429) | Prospective case series | 12 | PRP | None | Two intradermal/subdermal PRP injections (6-weeks intervals) | 12 weeks | Histopathological and clinical assessment (VAS and IGA) | PRP reduced histopathological inflammation in women with vulvar lichen sclerosus without the potential adverse effects associated with topical or systemic immunomodulators | IV |
| Lichen sclerosus | Navarrete et al. (430) | Prospective case series | 5 | PRP | None | PRP infiltration at 8-weeks intervals | 12 months | Clinical assessment (IGA and DLQI) | PRP appears to be safe and effective in controlling symptoms and improving quality of life, although its effects on visible clinical changes appear to be limited. | IV |

*Continued on the next page*

***Table S1.*** *Continued*

| Lichen sclerosus | Boero et al. (431) | Prospective case series | 10 | CB-PRP | None | Three injections at monthly intervals | 6 months | Vulvoscopy, vulvar elasticity and color, clinical assessment and patient’s satisfaction (NRS, FSFI, HADS and SF-12), and adverse events | CB-PRP appears to be a promising treatment for vulvar lichen sclerosus. Available evidence suggests that it is safe and may improve symptoms and sexual function | IV |
| --- | --- | --- | --- | --- | --- | --- | --- | --- | --- | --- |
| Lichen sclerosus | Boero et al. (432) | Prospective self-controlled study | 50 | PRP (50) | Corticosteroids (50) | Three PRP infiltrations (4 to 6-weeks intervals) | 9 months | Vulvoscopy, vulvar elasticity and color, clinical assessment and patient’s satisfaction (NRS, FSFI, HADS and SF-12), and adverse events | Following standard medical therapy, PRP may improve symptoms of vulvar lichen sclerosus while also promoting functional recovery | II |
| Lichen sclerosus | Tedesco et al. (433) | Prospective case series | 31 | PRP | None | Three PRP infiltrations (15-day intervals) | 12 months | Clinical assessment and side effects | PRP is a simple, safe, and immunologically biocompatible treatment that promotes tissue repair. However, further controlled studies are needed to better define its role in lichen sclerosus | IV |
| Lichen sclerosus | Tedesco et al. (434) | Prospective case series | 94 | PRP | None | Three PRP infiltrations (15-day intervals) | 6 months | Clinical assessment and patient’s satisfaction (DLQI) | PRP-based therapy may improve quality of life and sexual function in patients with lichen sclerosus while highlighting gender-related differences in symptom severity and age at disease onset | IV |
| Lichen sclerosus | Tedesco et al. (435) | Prospective case series | 6 | PRP | None | Single PRP infiltration | 1 month | Clinical assessment and video thermography (VTG) | PRP appears to be an effective treatment for vulvar lichen sclerosus, and videothermography may serve as a useful adjunctive tool for disease identification and treatment follow-up | IV |

*Continued on the next page*

***Table S1.*** *Continued*

| Lichen sclerosus | Medina-Garrido et al. (436) | Prospective case series | 28 | PRP | None | Three PRP infiltrations (6-weeks intervals) | 12 months | Clinical assessment (CSS) and patient’s satisfaction | PRP may provide symptom relief in selected patients with lichen sclerosus who are refractory to first-line therapy | IV |
| --- | --- | --- | --- | --- | --- | --- | --- | --- | --- | --- |
| Lichen sclerosus | Posey (437) | Prospective case series | 38 | PRP | None | Unknown | Unknown | Histopathological assessment and patients’s satisfaction | PRP failed to demonstrate efficacy in alleviating the symptoms of lichen sclerosus | IV |
| Lichen sclerosus | Paganelli et al. (438) | Retrospective observational cohort study | 46 | PRP, tCS, tCNI, tVitE, suppl, PhotoTp, Syst TT, SVF | None | Not reported | 2 years | Clinical assessment and patient’s satisfaction | The findings suggest that alternative therapies may have a role in the management of lichen sclerosus, although further research is needed to clarify its pathogenesis and optimize treatment | IV |
| Lichen sclerosus | Franíc et al. (439) | Retrospective case report | 1 | PRP | None | Two subdermal infiltrations (2-months interval) | 4 months | Patient’s satisfaction (ICIQ-VS) and histopathological assessment | PRP shows promise as a treatment for lichen sclerosus, but further evidence from RCTs is needed |  |
| Lichen sclerosus | Tedesco et al. (440) | Retrospective observational cohort pilot study | 319 | PRP, ADSCs, PRP + ADSCs | None | Three PRP infiltrations at 15-day intervals + additional treatments administered when needed after at least one year. ADSC-based grafting involved 2-3 day-surgery (4 months apart) | 1-9 years | Clinical assessment and patient’s satisfaction | The findings suggest that regenerative therapies, including PRP and ADSCs, may improve disease control in symptomatic lichen sclerosus patients who do not respond to conventional treatment and could potentially reduce the risk of neoplastic progression. However, further studies are needed to confirm their role in cancer prevention | IV |
|  |  |  |  |  |  |  |  |  |  |  |

*Continued on the next page*

***Table S1.*** *Continued*

| Lichen sclerosus | Tedesco et al. (441) | Prospective single-arm pilot study | 40 | AD-SVF + PRP grafting | None | Two surgical procedures (4-months interval) | 6 months | Clinical assessment and patient’s satisfaction (DLQI) | Clinical results support the preferential use of combined AD-SVF and PRP therapy in patients with early-stage disease, suggesting a synergistic effect. In contrast, this combination does not appear to be beneficial and is therefore discouraged in patients with late-stage disease | IV |
| --- | --- | --- | --- | --- | --- | --- | --- | --- | --- | --- |
| Vitiligo | Feng et al. (442) | Systematic review and meta-analysis of RCTs | 7 RCTs involving 366 patients | FCO_2_ + PRP, fractional erbium-YAG laser + PRP (+ latanoprost) | FCO_2_ + NB‐UVB, FCO_2_ alone, latanoprost alone, FCO_2_ + latanoprost | Variable | Variable | Grade of repigmentation, patient’s satisfaction and adverse events | This meta-analysis suggests that combining PRP with fractional laser therapy is an effective and safe treatment for vitiligo, demonstrating superior efficacy to control interventions with comparable adverse event rates. However, larger, high-quality studies are needed to confirm these findings | I |
| Vitiligo | Wang et al. (443) | Systematic review and meta-analysis of clinical studies | 3 RCTs and 3 retrospective cohort studies involving 142 patients | PRP | Mainly conventional therapy, excimer laser and topical fibrinogen/thrombin | Variable | Variable | Grade of repigmentation, patient’s satisfaction and adverse events | Current evidence suggests that PRP is a promising and safe adjunctive treatment for vitiligo, improving repigmentation outcomes. However, larger, high-quality randomized controlled trials are needed to confirm its efficacy | II |
| Vitiligo | Khalili et al. (444) | RCT | 41 | FCO_2_ + excimer lamp + PRP | FCO_2_ + excimer lamp + Tacrolimus | Three PRP treatments (1-month intervals). The excimer lamp was used twice a week | 9 months | Grade of repigmentation, patient’s satisfaction and side effects | The combination of fractional CO₂ laser, 308-nm excimer lamp, and PRP appears to be an effective treatment for refractory vitiligo, particularly in difficult-to-treat acral areas | II |

*Continued on the next page*

***Table S1.*** *Continued*

| Vitiligo | Jafarzadeh et al. (445) | Systematic review of clinical studies | 26 RCTs, 15 single-arm prospective clinical studies, 5 non-randomized clinical studies and 2 case series involving 2186 patients | | FCO_2_ + PRP, PRP, PRP + excimer laser, minipunch grafting, fractional erbium-YAG laser + PRP, MKTP, MKTP + PRF, ECS + FCS, NCORSHFS , EMT, HFMT, NCES, MSCs and regenerative therapies | | Mainly FCO_2_, excimer laser, microneedling and regenerative therapies | Variable | Variable | Clinical assessment, patient’s satisfaction and adverse events | Regenerative medicine appears to be a safe and effective treatment for vitiligo and may be used alone or in combination with other therapies. Further clinical trials are needed to evaluate emerging regenerative approaches and strengthen the evidence base | II |
| --- | --- | --- | --- | --- | --- | --- | --- | --- | --- | --- | --- | --- |
| Vitiligo | Chen et al. (446) | Systematic review and meta-analysis of RCTs | 11 RCTs involving 670 patients | Excimer laser/FCO_2_/NCES /NB‐UVB/vitD + PRP | | Excimer laser, FCO_2_, NCES, NB‐UVB, vitD | | Variable | Variable | Grade of repigmentation, patient’s satisfaction and adverse events | PRP combined with 308-nm excimer laser or fractional CO₂ laser is a safe and effective treatment for vitiligo, improving repigmentation | I |
| Vitiligo | Chen et al. (447) | Systematic review and meta-analysis of RCTs | 6 RCTs involving 302 patients | PRP + excimer laser | | Excimer laser (+ tacrolimus) | | Variable | Variable | Grade of repigmentation and adverse events | This meta-analysis supports the combined use of PRP and 308-nm excimer laser therapy for vitiligo, demonstrating superior efficacy compared with monotherapy | I |
| Vitiligo | Omar et al. (448) | RCT | 60 | Latanoprost + FCO_2_ + PRP (20) | | Latanoprost (20) Latanoprost + FCO_2_ (20) | | 2-week interval for 3 months | 4 months | Clinical and histopathological assessment | FCO₂ laser combined with PRP enhances the effectiveness of topical latanoprost in treating localized stable vitiligo, resulting in greater and faster repigmentation than latanoprost alone | II |
| Vitiligo | Mercuri et al. (449) | Review of clinical studies (systematic search) | 5 RCTs and 1 prospective single-arm pilot study | LA-PEEST + fractional erbium-YAG laser/NCES /NB‐UVB/FCO_2_ + PRP | | LA-PEEST + fractional erbium-YAG laser, NCES, NB‐UVB, FCO_2_, laser ablation | | Single procedure | 6 months | Clinical assessment, grade of repigmentation and adverse events | PRP-enriched epidermal suspension transplantation may enhance healing and repigmentation in vitiligo, although larger controlled studies are needed to confirm its efficacy | II |

*Continued on the next page*

***Table S1.*** *Continued*

| Vitiligo | Zhu et al. (450) | RCT | 20 | PRP + excimer phototherapy (30) | Excimer phototherapy (20) | Five PRP intradermal injections (1-month intervals) | 5 months | Grade of repigmentation and *in vitro*/*in vivo* transcriptomic analysis of DMPs | PRP provided additional clinical benefits in acral vitiligo by enhancing repigmentation and may represent a promising therapeutic strategy for treatment-resistant disease | II |
| --- | --- | --- | --- | --- | --- | --- | --- | --- | --- | --- |
| Vitiligo | Abdel-Halim et al. (451) | RCT | 15 | NCECS + PRP (15) | No treatment (15) | Single procedure | 3 months | Lesion extent and pigmentation | NCECS in PRP did not improve surgical outcomes in acral vitiligo or vitiligo overlying joints compared with standard treatment | II |
| Vitiligo | Mercuri et al. (452) | Prospective single-arm pilot study | 27 | Monocyte-rich PRP + 1927 nm fraxel laser and 308 nm excimer laser | None | Nine sessions in 80 days | 10 months | Clinical assessment (VES and grade of re-pigmentation) | This pilot study suggests that monocyte-rich PRP combined with laser therapy may provide sustained clinical improvement in vitiligo, with therapeutic benefits maintained for up to 10 months | IV |
| Vitiligo | Yin et al. (453) | Retrospective case report | 1 | NB-UVB + PRP | None | Three PRP treatments (1-month intervals). NB-UVB administered 3 times a week | 3.5 months | Grade of re-pigmentation | This case supports PRP as a promising adjunct to phototherapy for vitiligo, although larger prospective controlled studies are needed to establish its efficacy | IV |
| Vitiligo | Salem et al. (454) | Prospective self-controlled study | 17 | PRP + MPG + phototherapy (17) | MPG + phototherapy (17) | Three PRP treatments (1-month intervals) | 20 weeks | Grade of re-pigmentation, lession surface and adverse events | PRP accelerated repigmentation when combined with MPG and phototherapy but did not improve the final treatment outcome | II |
| Vitiligo | Rekik et al. (455) | Prospective case series | 10 | PRP | None | Variable (1-5 sessions of intradermal injections) | 6 months | Clinical assessment (number of lessions to have enhancement, color enhancement and regression degree) and side effects | PRP is a safe and promising treatment option for stable vitiligo lesions across different body sites, with minimal adverse effects | IV |

*Continued on the next page*

***Table S1.*** *Continued*

| Vitiligo | Ibrahim et al. (456) | Prospective self-controlled pilot study | 60 | NB-UVB + PRP (60) | NB-UVB (60) | Eight intradermal PRP injections (2-weeks intervals) | 7 months | Clinical and histopathological assessment | Intradermal PRP combined with NB-UVB may enhance vitiligo treatment by reducing the duration of phototherapy while maintaining a safe and well-tolerated profile; however, longer-term studies are needed | II |
| --- | --- | --- | --- | --- | --- | --- | --- | --- | --- | --- |
| Alopecia areata | Anitua et al. (457) | Systematic review and meta-analysis of RCTs | 9 RCTs involving 433 patients | PRP | ILC, PBO | Variable | Variable | Hair density, SALT score, patient’s satisfaction, clinical improvement, hair loss and side effects | Moderate evidence supports PRP as a safe and effective therapy for improving hair density, reducing hair loss, and increasing patient’s satisfaction. However, its benefits on hair thickness and follicular parameters remain uncertain, highlighting the need for standardized, high-quality trials | I |
| Alopecia areata | Tejapira et al. (458) | Systematic review of clinical studies | 11 RCTs, 4 non-randomized and 17 case series/reports | PRP | Mainly PBO, ILC, minoxidil | Variable | Variable | Clinical improvement, hair regrowth, SALT score, patient’s satisfaction and side effects | PRP shows promise for alopecia areata and primary cicatricial alopecias, particularly in patients with poor response to conventional therapies. However, standardized protocols and stronger evidence are needed to establish its role, especially in inflammatory-mediated alopecias | II |
| Alopecia areata | Cruciani et al. (460) | Systematic review and meta-analysis of RCTs | 9 RCTs involving 404 patients | PRP | ILC, PBO | Variable | Variable | Hair density, hair count, hair regrowth and adverse events | Evidence for the efficacy of PRP in treating alopecia remains limited and is largely based on low-quality studies | I |
| Alopecia areata | Rinaldi et al. (461) | RCT | 60 | PRP (30) | PBO (30) | Twice a week for 3 months (topical) | 120 days | Clinical improvement, SALT score and patient’s satisfaction | PRP-like cosmetics could represent a safer alternative to autologous PRP for alopecia areata, although further studies are needed to confirm its efficacy | II |
| Alopecia areata | Vazquez et al. (468) | Retrospective case report | 1 | aPRF | None | Two intralesional sessions (2-months intervals) | 6 months | Hair regrowth | This case suggests that micronized injectable aPRF may represent a promising therapeutic option for alopecia areata following COVID-19 infection | IV |

*Continued on the next page*

***Table S1.*** *Continued*

| Alopecia areata | Todorova et al. (469) | Retrospective case report | 1 | PRP | None | Three intradermal sessions (1-month intervals) | 2 years | SALT score, patient’s satisfaction (DLQI ) and trichoscopy | This report presents the case of a woman with extensive patchy alopecia areata who achieved successful treatment outcomes with PRP therapy | IV |
| --- | --- | --- | --- | --- | --- | --- | --- | --- | --- | --- |
| Alopecia areata | Singh et al. (471) | Prospective case series | 20 | PRP | None | Six intradermal sessions (4-weeks intervals) | 1 year | Hair regrowth and side effects | PRP may have a beneficial role in the treatment of alopecia areata. However, further studies with larger cohorts and long-term follow-up are required to validate these findings and determine its efficacy, particularly given the high recurrence rates commonly observed in this condition | IV |
| Alopecia areata | Suh et al. (472) | Retrospective case series | 2 | PRP | None | Variable (1 to 2-month intervals) | Variable (up to 2 years) | Clinical improvement and patient’s satisfaction | PRP showed promising short-term improvement in inflammation in two patients with refractory folliculitis decalvans, despite the limitations of a small case series and temporary effects with subsequent relapse. Larger randomized studies are needed to confirm its long-term efficacy, optimize treatment parameters, and define its role in scarring alopecias. | IV |
| Hidradenitis suppurativa | Gierek et al. (473) | Retrospective comparative cohort study | 61 | Local skin flaps + PRP (23) | Local skin flaps (26) Co-graft ADM + STSG (7) STSG (5) | Four PRP injections after surgery (days 0, 1, 2, and 3 ) | 6 months | Wound healing (LASCA), frequency of complications and time of hospitalization | PRP injections into skin flaps, combined with acellular dermal matrix co-grafting and split-thickness skin grafts, represent promising surgical options for the management of hidradenitis suppurativa | III |

*Continued on the next page*

***Table S1.*** *Continued*

| Hidradenitis suppurativa | Nicoli et al. (474) | Retrospective case report | 1 | PRP gel + HMA | None | Single procedure (topical + perilesional injections) | 1 year | Clinical assessment and patient’s satisfaction | Severe hidradenitis suppurativa can be safely and effectively treated with wide excision. The use of PRP gel combined with HMA represents a novel, minimally invasive adjunct that is easy to apply, well tolerated, and associated with favorable outcomes, offering a promising alternative approach | IV |
| --- | --- | --- | --- | --- | --- | --- | --- | --- | --- | --- |
| Hidradenitis suppurativa | Vossen et al. (475) | Retrospective case series | 4 | SSG + PRP | None | Single procedure (topical ) | 9 months | Wound healing | Wide excision with topical PRP and SSG achieved complete healing and disease resolution in advanced hidradenitis suppurativa, suggesting PRP is a useful adjunct to surgical treatment | IV |
| Hidradenitis suppurativa | Edek et al. (476) | Retrospective case report | 1 | SSG + PRF | None | Single procedure (topical ) | 6 months | Wound healing and recurrence | The use of PRF as an adjunct after hidradenitis suppurativa surgery may accelerate wound healing and improve postoperative recovery | IV |
| Acne | Cruciani et al. (478) | Systematic review of systematic review | 10 RCTs, 21 split-face studies and 3 uncontrolled studies | PRP, PRP + MN, PRP + FCL | Mainly saline, MN + saline, FCL, MN | Variable | Variable | Clinical improvement, patient’s satisfaction and adverse events | Current evidence is insufficient to support the routine use of PRP combined with microneedling or laser therapy for acne scars, highlighting the need for well-designed studies to establish its efficacy | I |
| Acne | Moftah et al. (479) | Randomized split-face comparative study | 30 | PRP (30) | pulsed Nd:YAG (30) | Three PRP infiltrations (2-weeks intervals) | 2 months | Clinical improvement (lesion counting and grading according to Hayashi score), patient’s satisfaction (VAS), recurrence and side effects | Both intralesional PRP injections and 1064-nm long-pulsed Nd laser therapy are safe and effective treatments for reducing inflammatory and non-inflammatory acne vulgaris in adolescents and adults | II |

*Continued on the next page*

***Table S1.*** *Continued*

| Acne | Diab et al. (480) | Randomized split-face comparative study | 30 | PRP + MN (30) | PRF + MN (30) | Three PRP/PRF infiltrations (3-weeks intervals) | 3 months | Clinical improvement (GSGS and QGS) and patient’s satisfaction | Combining PRP or PRF with microneedling enhances treatment efficacy for acne scars, and fluid PRF appears to be a safe and effective alternative to PRP | II |
| --- | --- | --- | --- | --- | --- | --- | --- | --- | --- | --- |
| Acne | Ibrahim et al. (481) | Prospective split-face comparative study | 40 | PRP (40) | Erythromycin (40) | Six intradermal PRP sessions (2-weeks intervals) | 6 months | Clinical improvement, patient’s satisfaction and adverse events | PRP is a safe and effective treatment for inflammatory acne and may serve as an alternative to systemic therapies | III |
| Acne | Kang et al. (483) | Systematic review and meta-analysis of clinical studies | 4 RCTs and 10 split-face non-randomized controlled studies involving 472 patients | PRP + MN | MN | Variable | Variable | Clinical improvement, patient’s satisfaction and adverse events | MN combined with PRP is more effective than MN alone for the treatment of acne scars | I |
| Acne | Meghe et al. (484) | Scooping review (systematic literature search) | 4 RCTs and 10 split-face non-randomized controlled studies | PRP + MN | MN | Variable | Variable | Clinical improvement (GQS) and patient’s satisfaction | Combination therapy with PRP improves clinical outcomes, although standardized PRP preparation protocols are required | I |

*Continued on the next page*

***Table S1.*** *Continued*

| Acne | Aljefri et al. (485) | Systematic review and meta-analysis of RCTs | 11 RCTs involving 313 patients | PRP + FCO_2_ | FCO_2_ | Variable | Variable | Clinical improvement (GBS), patient’s satisfaction and adverse events | Combined laser therapy and PRP is a safe and effective treatment for moderate to severe atrophic acne scars, with synergistic therapeutic effects | I |
| --- | --- | --- | --- | --- | --- | --- | --- | --- | --- | --- |
| Acne | Roohaninasab et al. (487) | RCT | 15 | PRP + HA (15) | PRP (5) HA (5) PBO (5) | Two sessions | 3 months | Colorimetry, tewametry, corneometry, erythema, melanin indices, patients and physicians satisfaction | The combination of PRP and non-cross-linked hyaluronic acid is a promising treatment for atrophic acne scars, showing greater efficacy than either therapy alone. Further studies are needed to confirm these findings and optimize treatment protocols | II |
| Acne | Alegre et al. (488) | Retrospective case series | 9 | PRGF-gel + fractional ablative laser | None | 1-4 infiltrations (4 to 6-weeks intervals) | 6 months | Clinical assessment (ECCA), patient’s satisfaction and adverse events | Combined PRGF gel and fractional ablative laser therapy may improve post-acne scars and promote overall skin rejuvenation | IV |
| Acne | Ebrahimi et al. (489) | Systematic review and meta-analysis of RCTs | 23 RCTs (13 enrolled in meta-analysis) | PRP, PRP + MN, PRP + FCL | Mainly saline, MN + saline, FCL, MN | Variable | Variable | Clinical improvement, patient’s satisfaction and adverse events | PRP is a safe and effective treatment for atrophic scars and enhances the efficacy of ablative lasers and microneedling while reducing treatment-related adverse effects | I |
| Rosacea | Ghoz et al. (490) | RCT | 40 | PRP (40) | PPP (40) | Six intradermal PRP sessions (2-weeks intervals) | 6 months | Clinical evaluation (RGS, percentage of improvement and GAIS) patient’s satisfaction and histopathological assessment and side effects | PRP is a safe and effective treatment for rosacea and may serve as an alternative to systemic therapies, particularly when they are contraindicated | III |
| Rosacea | Hwang(491) | Retrospective case series | 3 | PRP enriched with CD34+ stem cells | None | Not reported | 3 months | Clinical improvement and patient’s satisfaction | PRP enriched with peripheral blood CD34+ stem cells may provide additional therapeutic benefits in rosacea, although long-term prospective studies are needed to confirm its efficacy | IV |

*Continued on the next page*

***Table S1.*** *Continued*

| Rosacea | Pang et al. (492) | Retrospective case series | 5 | PRP | None | Single intradermal session | 1 month | Clinical assessment | PRP, alone or combined with other therapies, is a promising treatment for rosacea, with the potential to improve outcomes and overcome limitations of conventional therapies. Further studies are needed to confirm its efficacy and optimize treatment protocols | IV |
| --- | --- | --- | --- | --- | --- | --- | --- | --- | --- | --- |
| Infectious dermatoses | Lu et al. (493) | Systematic review and meta-analysis of RCTs | 7 RCTs involving 568 patients | PRP + PRF, PRP, PRF | Pulsed radiofrequency | Variable | Variable | NRS, sleep quality, rescue medication dosage and adverse events | Combined PRF–PRP therapy is more effective than conventional pulsed radiofrequency for treating herpes zoster neuralgia, improving sleep quality and reducing postoperative analgesic requirements | I |
| Infectious dermatoses | Kan et al. (494) | Scooping review (systematic literature search) | 17 RCTs, 5 single-arm studies, and 2 retrospective studies | PRP, PRF, PRP + PRF, NB + PRF, PRP + IFN-α2b | Drugs, NB, PRF, IFN-α2b, corticosteroids | Variable | Variable | Pain score, sleep quality, DLQI, serum indices and analgesic dosage | PRP shows potential benefits for the treatment of zoster-associated pain, but current evidence remains limited, and further high-quality clinical studies are needed to confirm its efficacy | II |
| SCARs | Klama-Baryla et al. (495) | Retrospective case report | 1 | Standard care + biostatic human amnion transplant + PRP | None | PRP used to cover wounds after two surgical interventions (topical) | Not reported | Wound healing | Topical PRP combined with biostatic human amnion may be a promising treatment for toxic epidermal necrolysis/Stevens–Johnson syndrome | IV |
| AIBDs | Mercuri et al. (496) | Retrospective case report | 1 | PRP | None | Five injections (2-months intervals) | 6 months | Clinical improvement, patient’s satisfaction and adverse events | Photoactivated low-temperature PRP showed promising results in treating adult-onset linear morphea en coup de sabre, with marked local improvement and high patient’s satisfaction in a case report | IV |

*Continued on the next page*

***Table S1.*** *Continued*

| AIBDs | Ibrahim et al. (497) | Prospective single-arm pilot study | 5 | PRP | None | 12 PRP injections (weekly) | 12 weeks | Clinical improvement (LoSCAT, LoSSI and LoSDI) and side effects | PRP is a promising treatment for localized morphea, providing favorable cosmetic outcomes with minimal adverse effects | IV |
| --- | --- | --- | --- | --- | --- | --- | --- | --- | --- | --- |
| AIBDs | El-Komy et al. (498) | Prospective single-arm pilot study | 7 | PRP | None | Weekly to monthly PRP injections | 12 months | Clinical assessment and patient’s satisfaction (PDAI, pain score and eating/drinking discomfort) | PRP injections may accelerate healing and reduce pain and eating discomfort associated with oral erosions and ulcers in pemphigus vulgaris | IV |
| AIBDs | Tiwari et al. (499) | Prospective single-arm pilot study | 15 | PRP | None | Three perilesional injections (weekly) | Not reported | Clinical assessment (POLIS) | Intralesional PRP may be an effective treatment for refractory oral ulcers in pemphigus vulgaris, particularly when corticosteroids are ineffective or contraindicated | IV |
| AIBDs | Bardazzi et al. (500) | Retrospective case report | 1 | PRP | None | Unknown | Unknown | Clinical improvement and patient’s satisfaction | Adjunctive PRP resulted in clinical improvement of refractory oral lesions in a patient with pemphigus vulgaris, supporting its potential as an adjuvant therapy | IV |
| AIBDs | Gabusi et al. (501) | Retrospective case series | 1 | PRP | None | Weekly PRP injections for 2 months | 1 year | Clinical improvement (ODSS ) and pain (VAS) | PRP is a safe and promising adjunctive treatment for refractory gingival lesions in AIBDs, although randomized controlled trials are needed to confirm its efficacy | IV |
| AIBDs | Anitua et al. (502) | Retrospective case report | 1 | PRGF | None | Four PRGF infiltrations (weekly) | 13 months | Clinical assessment and pain (VAS) | PRGF may be a useful adjunctive therapy for the management of extensive oral lesions in mucous membrane pemphigoid | IV |
| AIBDs | Nopriyati et al. (503) | Retrospective case report | 1 | PRP | None | Topical PRP for 7 days (from 7th to 14th day) | Not reported | Clinical improvement and patient’s satisfaction | PRP may improve clinical outcomes in patients with pemphigus foliaceus, although further studies are needed to confirm its efficacy | IV |

*Continued on the next page*

***Table S1.*** *Continued*

| AIBDs | El-Komy et al. (504) | Prospective single-arm pilot study | 11 | PRP | ILC | PRP injections every 14 days for 3 months | 3 months | Clinical assessment and patient’s satisfaction (PDAI and pain score) | Autologous PRP may be an effective treatment for refractory oral erosions in pemphigus vulgaris, particularly when intralesional corticosteroids are contraindicated | III |
| --- | --- | --- | --- | --- | --- | --- | --- | --- | --- | --- |
| AIBDs | Imam et al. (505) | Retrospective case report | 1 | ILC + PRP | None | Three sessions in 4 months | 4 months | Clinical improvement, patient’s satisfaction and histopathological assessment | PRP may be a promising adjunctive therapy for inflammatory alopecia, underscoring the importance of multidisciplinary management of lupus and further investigation into novel therapeutic approaches for alopecia | IV |
| AIBDs | Polster et al. (506) | Retrospective case report | 1 | PRP | None | Three injections (3-months intervals) | 1 year | Clinical improvement, hair regrowth and patient’s satisfaction | Three sessions of PRP injections resulted in significant hair regrowth, including in areas of scarring alopecia | IV |
| AIBDs | Chikhalkar et al. (507) | Case report with intra-patient control | 1 | iPRF (1) | ILC (1) | Three sessions (3-weeks intervals) | 3 months | Hair regrowth | PRF showed promising results in reversing hair loss associated with discoid lupus erythematosus in a case report | IV |
| AIBDs | Huber et al. (257) | Prospective single-arm pilot study | 6 | P-PRP | None | Six periumbilical subcutaneous applications (2-weeks intervals) + three periumbilical subcutaneous applications applications (1-month intervals) | 1 year | Clinical improvement and patient’s satisfaction ( BR-BDCAF and SF-36) | P-PRP promoted an anti-inflammatory profile and improved oral ulcer outcomes in patients with patients with Behçet's disease | IV |
| Neuropathic pain | Anitua et al. (508) | Systematic review of RCTs | 12 RCTs involving 754 patients | PRP | Mainly PBO, corticosteroid, pregabalin PO, conventional therapy | Variable | Variable | Pain intensity, quality of life, nerve repair, wound healing, symptom severity and functional status of patients | PRP may be an effective treatment for neuropathic pain across different etiologies, although further studies are needed to confirm its efficacy | I |

*Continued on the next page*

***Table S1.*** *Continued*

| Neuropathic pain | El-Hamd et al. (509) | Prospective single-arm pilot study | 45 | PRP | None | PRP injections every 2 weeks for 2 months | 5 months | Clinical improvement and patient’s satisfaction (VAS, NRS, VRS and SF-36) | PRP is an effective treatment for patients with postherpetic neuralgia | IV |
| --- | --- | --- | --- | --- | --- | --- | --- | --- | --- | --- |

******* *Levels of evidence were classified using an evidence hierarchy based on study design*

**Abbreviations:** *AD-SVF, adipose-derived stromal vascular fraction; ADM, acellular dermal matrix; ADSC, adipose-derived stem cells; AIBDs, Autoimmune Blistering Diseases; APGR, autologous platelet gel-rich plasma; APRG, autologous platelet-rich gel; BR-BDCAF, Behçet's Disease Current Activity Form; CB-PRP, cord-blood platelet-rich plasma; CD34, cluster of differentiation 34; CSS, Clinical Severity Score; CTCAE, Common Terminology Criteria for Adverse Events; DLQI, Dermatology Life Quality Index; EASI, Eczema Area and Severity Index; ECCA,* Échelle d'Évaluation Clinique des Cicatrices d'Acné*; ECS, epidermal cell suspension; EMT, epithelialization time; EPUAP, European Pressure Ulcer Advisory Panel; FCL, fractional CO₂ laser; FCO₂, fractional carbon dioxide laser; FFASS, Fitzpatrick Facial Acne Scar Scale; FSFI, Female Sexual Function Index; FUE, follicular unit extraction; GAIS, Global Aesthetic Improvement Scale; GBS, Global Blister Score; GQS, Global Quartile Scale; GSGS, Global Scar Grading System; HA, hyaluronic acid; HADS, Hospital Anxiety and Depression Scale; HFMT, hair follicle mesotherapy; HMA, Hyalomatrix®; ICIQ-VS, International Consultation on Incontinence Questionnaire–Vaginal Symptoms; IFN-γ, interferon gamma; IGA, Investigator Global Assessment; IL-17, interleukin-17; ILC, intralesional corticosteroid; LA-PEEST, laser-assisted platelet-enriched epithelial sheet transplantation; LASCA, laser speckle contrast analysis; LPP, lichen planopilaris; LPPAI, Lichen Planopilaris Activity Index; MKTP, melanocyte–keratinocyte transplantation procedure; MN, microneedling; MPG, micrograft; MTX, methotrexate; NB-UVB, narrowband ultraviolet B; NCECS, non-cultured epidermal cell suspension; NCES, non-cultured epidermal cell suspension; NCORSHFS, non-cultured outer root sheath hair follicle suspension; NRS, Numeric Rating Scale; ODSS, Oral Disease Severity Score; P-PRP, pure platelet-rich plasma; PASI, Psoriasis Area and Severity Index; PBO, placebo; PDAI, Pemphigus Disease Area Index; PGA, Physician Global Assessment; PO; POLIS, Psoriasis Onycholysis Severity Index; PPP, platelet-poor plasma; PRF, platelet-rich fibrin; PRGF, plasma rich in growth factors; PRP, platelet-rich plasma; QGS, Quartile Grading Scale; RCT, randomized controlled trial; RGS, Rosenberg Global Scale; RTOG, Radiation Therapy Oncology Group; SALT, Severity of Alopecia Tool; SCARs, Severe Cutaneous Adverse Reactions; SF-12, 12-Item Short Form Health Survey; SF-36, 36-Item Short Form Health Survey; SSG, split-thickness skin graft; STSG, split-thickness skin graft; SVF, stromal vascular fraction; TT, trichoscopy test; UVB, ultraviolet B; VAS, visual analogue scale; VES, Vitiligo Extent Score; VISIA, VISIA Complexion Analysis System; VRS, Verbal Rating Scale; VTG, vacuum therapy graft; YAG, yttrium aluminum garnet*
